# Antibiotic Consumption in Danish Intensive Care Units, 2013–2023: A Nationwide Study of Temporal Trends

**DOI:** 10.1111/aas.70124

**Published:** 2025-09-22

**Authors:** Nick Frørup Meier, Frederik Boëtius Hertz, Anders Granholm, Anders Perner, Fredrik Sjövall, Kathrine Bruun Svan, Morten Hylander Møller, Marie Helleberg

**Affiliations:** ^1^ Department of Intensive Care Copenhagen University Hospital – Rigshospitalet Copenhagen Denmark; ^2^ Department of Clinical Microbiology Copenhagen University Hospital – Rigshospitalet Copenhagen Denmark; ^3^ Department of Immunology & Microbiology University of Copenhagen Copenhagen Denmark; ^4^ Section of Biostatistics, Department of Public Health University of Copenhagen Copenhagen Denmark; ^5^ Department of Intensive and Perioperative Care Skåne University Hospital Malmö Sweden; ^6^ The Hospital Pharmacy The Capital Region of Denmark Herlev Denmark; ^7^ Department of Clinical Medicine, Faculty of Health Sciences University of Copenhagen; ^8^ Department of Infectious Diseases Copenhagen University Hospital – Rigshospitalet Copenhagen Denmark; ^9^ Centre of Excellence for Health, Immunity and Infections Copenhagen University Hospital – Rigshospitalet Copenhagen Denmark

**Keywords:** antibiotic consumption, antibiotic resistance, antibiotic stewardship, antibiotics, antimicrobials, critical illness, intensive care unit

## Abstract

**Background:**

Antibiotics are widely used in intensive care units (ICUs), yet detailed nationwide data on ICU‐specific consumption are limited. In 2012, the Danish Health Authority introduced a policy framework to promote prudent antibiotic use. We evaluated national trends in antibiotic consumption across Danish ICUs from 2013 to 2023 considering this initiative.

**Methods:**

We conducted a nationwide observational study including all adult ICUs across 29 public hospitals in Denmark. All ICU admissions from January 1, 2013, to December 31, 2023, were included, covering 1,121,639 ICU patient days. Antibiotic consumption was assessed using defined daily doses (DDD) derived from national sales data. No interventions were implemented.

**Results:**

During the study period, a total of 1,624,281 DDD of intravenous antibiotics were administered. Overall antibiotic consumption declined from 1705 to 1348 DDD per 1000 patient days, representing a 21% relative reduction. Marked decreases were observed for fluoroquinolones (−80%), first‐ and second‐generation cephalosporins (−61%), and carbapenems (−34%). Conversely, consumption of penicillins with beta‐lactamase inhibitors increased by 139%. Run chart analyses indicated these trends were non‐random. Importantly, no deterioration in clinical outcomes was observed. Antibiotic consumption varied widely across ICUs and regions. According to both WHO's AWaRe framework and a modified national classification, ‘Watch’ antibiotics accounted for the largest share of consumption.

**Conclusion:**

In this nationwide study of Danish ICUs, antibiotic consumption decreased substantially over an 11‐year period—driven by reductions in broad‐spectrum classes—without evidence of worsening clinical outcomes. These data document a sustained decline in broad‐spectrum antibiotic use in Danish intensive‐care practice and may provide a benchmark for other high‐income healthcare systems.

**Editorial Comment:**

This inventory of antibiotics consumption in Danish intensive care units demonstrates a recent reduction in broad‐spectrum antibiotic ordering which differs from the well‐known increase of antibiotics used in health care in general.

## 1 Introduction

Increasing antimicrobial resistance is a global health concern and recognized as one of the most pressing challenges in modern medicine [1]. The overuse of antibiotics accelerates the development of resistant microorganisms, posing significant threats to public health worldwide [1, 2, 3, 4].

In the intensive care unit (ICU), approximately 70% of patients receive antibiotic treatment in recent years [2, 3]. Estimates indicate that average consumption of antibiotics in the ICU population reaches 1563 defined daily doses (DDD) per 1000 patient days, nearly three times the consumption rate observed in general wards, with notable differences, especially in the use of broad‐spectrum antibiotics [5]. Overuse of antibiotics in the ICU setting appears widespread, and it is estimated that up to 30% of ICU patients treated with antibiotics for presumed infections have a low likelihood thereof [6, 7].

Surveillance of antibiotic consumption within the ICU setting has revealed substantial insights into patterns of broad‐spectrum antibiotic use, including the prevalence of inappropriate prescribing, variations in consumption across institutions, and the impact of such use on antimicrobial resistance trends [8, 9, 10]. While initiatives such as the Danish Integrated Antimicrobial Resistance Monitoring and Research Program (DANMAP) and the European Centre for Disease Prevention and Control's (ECDC) surveillance efforts collect valuable data on antibiotic consumption across hospitals and primary care settings, they currently lack specific focus on ICUs. Mapping antibiotic use has helped guide stewardship efforts, set benchmarks, and inform policy [11, 12].

Generally, the consumption of antibiotics, particularly broad‐spectrum antibiotics, is rather low in the Danish health care sector [13, 14], but nationwide data on time trends in antibiotic use within ICUs have not been examined. Within the last decade, the Danish Health Authorities have introduced policy frameworks aimed at reducing the use of antibiotics and reductions in the use of cephalosporins, carbapenems, and fluoroquinolones. In contrast, international reports from comparable ICUs in Germany, Switzerland, and Sweden have shown stable or increasing antibiotic consumption trends over similar periods [8, 10, 15, 16]. We aimed to describe time trends in antibiotic consumption in Danish ICUs from 2013 to 2023.

The primary objective was to examine trends in total antibiotic consumption and its distribution by antibiotic class over the study period. Secondary objectives were to assess regional and ICU‐level variation in use, evaluate changes in the proportional use of antibiotics by WHO and adapted AWaRe classifications, and explore whether shifts in consumption coincided with changes in key clinical outcomes.

We hypothesized that antibiotic consumption within the Danish ICU setting had increased over the study period and that there would be notable variations in antibiotic consumption between regions and ICUs.

## 2 Materials and Methods

### 2.1 Study Design and Setting

This was a nationwide observational study analyzing data on intravenous antibiotic consumption from all ICUs across all five regions in Denmark from January 1, 2013, to December 31, 2023. We prepared this manuscript according to the Strengthening the Reporting of Observational Studies in Epidemiology (STROBE) guidelines [17] (S1).

We included data from 37 ICUs from 29 public hospitals in Denmark. We excluded 3 pediatric ICUs. ICUs were categorized based on their geographical region in Denmark, including both specialized and mixed medical‐surgical units across university and non‐university hospitals. In Denmark, only public hospitals have ICUs; therefore, this study covers all ICUs in Denmark. To enable temporal comparisons, ICUs within certain university hospitals were grouped into units corresponding to their respective hospitals (S2).

Ethical approval was not required for this type of study according to national authorities.

### 2.2 Data Collection and Management

We utilized antibiotic sales data and throughout the manuscript, the term ‘antibiotic consumption’ denotes sales‐derived DDD/1000 patient‐days and therefore serves as a proxy for bedside administration. This method has previously been used in Danish ICUs [18] and validated internationally [5, 19]. In Denmark, every antibiotic purchased and distributed to the ICUs is registered in the Register of Pharmaceutical Sales, which is run by Amgros I/S, a company owned by the Danish Regions. Data on monthly antibiotic sales were obtained from the register for each ICU from January 1, 2013, to December 31, 2023. Data included every antibiotic under the Anatomic Therapeutic Classification (ATC) code J01 (systemic antibacterials) at the fifth‐digit level, administration form, dose, package size, and DDD. This study focused exclusively on intravenously administered antibacterial drugs.

Number of yearly ICU admissions and length of ICU stays were obtained from the Danish Intensive Care Database [20]. We collected data on the number of medical, acute surgical, and elective surgical admissions per ICU and Simplified Acute Physiology Score (SAPS) III [21] for the year 2023, as this was the last year of the study period, and previous years had incomplete data. ICU bed counts for 2023 were obtained from hospital planning departments (S3). To explore whether changes in antibiotic consumption coincided with changes in clinical measures, we extracted data on key outcomes (30‐day mortality, median ICU length of stay, and proportions of patients requiring mechanical ventilation, inotropes, or dialysis) from the Danish Intensive Care Database (DID's) published annual reports, where available.

### 2.3 Definitions and Classifications

#### 2.3.1 Antibiotic Consumption

We calculated antibiotic consumption as DDDs per 1000 patient days [22]. Patient days were defined as total ICU inpatient care days over a period.

#### 2.3.2 WHO and Adapted WHO AWaRe Classification

We assessed antibiotic consumption according to the World Health Organization (WHO) Access, Watch, and Reserve (AWaRe) classification (S4) [23]. We also assessed antibiotic use using a Danish‐adapted AWaRe classification to reflect local guidelines [24].

#### 2.3.3 Antibiotic Grouping

We defined antibiotic groups based on the fourth level ATC code, that is, (1) penicillins, (2) combinations of penicillins with beta‐lactamase inhibitors (BL/BLI), (3) first‐ and second‐generation cephalosporins, (4) third‐ and fourth‐generation cephalosporins, (5) carbapenems, (6) fluoroquinolones, (7) glycopeptide antibacterials, and (8) imidazole derivatives. A ninth group, “other,” combined ten fourth‐level ATC categories with the lowest consumption rates (S5).

### 2.4 Statistics

We primarily used descriptive statistic. Categorical data were reported as numbers and percentages, and continuous data as medians with interquartile ranges (IQRs). Results were secondarily stratified by region and antibiotic grouping.

### 2.5 Run Charts

We evaluated antibiotic consumption and patient outcome trends using run charts [25, 26], a method of statistical process control assessing if there is evidence of time trends not being random. These charts visually display data over time, with the *x*‐axis representing the timeline and the *y*‐axis indicating measured quality indicators, including antibiotic consumption, 30‐day mortality, median ICU length of stay, and proportions of invasive mechanical ventilation, inotrope use, and dialysis. Each chart includes a median line. Random variation appears as points scattered around the median, whereas consistent trends appear as non‐random patterns, indicating statistically significant changes [25]. Parameters calculated for each chart included the median, useful data points, longest run, frequency of median crossings, and prediction limits per Anhøj [25, 26]. Non‐random variation (at the 5% statistical significance level) was identified when the longest run exceeded upper limits or crossings fell below lower prediction limits [25].

### 2.6 Missing Data

Missing data were imputed using the *next observation carried backward* method, with the mean calculated from the next four available observations [27]. All data management and calculations were performed in R (R Core Team, R Foundation for Statistical Computing, Vienna, Austria) version 4.4.2, primarily using the *tidyverse* packages [28].

## 3 Results

We included data from all Danish ICUs covering 1,121,639 patient days and a total of 1,624,281 DDDs. Characteristics of the ICUs in each region are presented in Table 1 (see S6 for further details). In 2023, ICUs had 340 beds in total (median 8; IQR 6–11; range 2–20). Most were mixed ICUs (84%). Eleven hospitals (38%) had an oncology department, and eight hospitals (28%) a haematology department, and 62% of ICUs were in non‐university hospitals. The median SAPS III across the units in 2023 was 57 (IQR: 45–69; range: 31–77). The median number of ICU admissions was 29,008 (IQR 26,930–29,654). The median ICU length of stay was 4 days (IQR 3–5) in 2013 and 3 days (IQR 3–4) in 2023 S7. The proportion of missing data is presented in S8.

**TABLE 1:** ICU and hospital characteristics.

Region	Number of ICUs, number	ICU characteristics per 2023						Hospital characteristics per 2023	
		Intensive care unit beds^a^	Admissions to intensive care unit^a^ ^,^ ^b^			Median SAPS III score^a^ ^,^ ^c^	Hospital size^a^	Oncology department^d^, number (%)	Haematological department^d^, number (%)
			Medical	Surgical					
				Acute	Elective				
Denmark	29	8 (6–11) [2–20]	62% (51%–79%) [4%–100%]	26% (18%–37%) [0%–84%]	3% (0%–9%) [0%–60%]	58 (53–61) [43–67]	316 (181–557) [19–905]	12 (41%)	8 (28%)
Capital region of Denmark	8	11 (6–13) [2–20]	78% (34%–79%) [4%–92%]	21% (19%–41%) [8%–84%]	0% (0%–0%) [0%–60%]	61 (59–63) [56–67]	508 (159–652) [74–905]	3 (38%)	1 (13%)
Region Zealand	5	6 (6–7) [6–9]	83% (55%–87%) [52%–92%]	13% (5%–28%) [0%–44%]	0% (0%–0%) [0%–3%]	63 (59–63) [58–65]	338 (213–340) [205–400]	1 (20%)	1 (20%)
The North Denmark Region	3	7 (6–9) [5–10]	60% (36%–69%) [26%–100%]	25% (19%–37%) [0%–43%]	7% (3%–25%) [0%–54%]	49 (45–54) [43–59]	66 (43–312) [19–558]	1 (33%)	1 (33%)
Central Denmark Region	6	8 (7–15) [4–20]	62% (56%–73%) [51%–92%]	34% (22%–35%) [0%–45%]	4% (3%–6%) [0%–11%]	54 (52–54) [50–57]	264 (201–499) [32–850]	3 (50%)	2 (33%)
The Region of Southern Denmark	7	10 (6–11) [3–20]	56% (39%–60%) [9%–85%]	33% (26%–37%) [11%–40%]	9% (4%–30%) [3%–53%]	57 (54–59) [51–59]	302 (195–343) [70–623]	3 (43%)	3 (43%)

*Note:* The table presents the characteristics of ICUs in Denmark for 2023, categorized by region. It includes the number of ICUs, ICU bed capacity, the distribution of admissions by medical, acute surgical, and elective surgical cases (as percentages of total admissions), and the median of median SAPS III scores (with IQR). Additionally, the table details hospital size (median number of beds with IQR) and the presence of oncology and haematology departments. See S6 for detailed ICU and hospital characteristics.

Abbreviations: %, percentage; ICU, intensive care unit; IQR, interquartile range; *n*, number; SAPS III, simplified Acute Physiology Score 3.

^a^ Data are presented as the median (interquartile range) and [range].

^b^ Missing data for admission type ranged from 0% to 20% (S8).

^c^ Missing data for SAPS III ranged from 0% to 100% (S8).

^d^ Indicates whether the hospital has an oncology (yes/no) or a haematology department (yes/no) (S3 and S6).

### 3.1 Time Trends in Total Antibiotic Consumption

#### 3.1.1 Overall Antibiotic Consumption

In total, 41 unique antibiotics were used (S5). The mean antibiotic consumption during the study period was 1474 DDD/1000 patient days and declined relatively by 21% over the study period, decreasing from 1705 to 1348 DDD/1,000 patient days (Table 2 and S9).

**TABLE 2:** National and regional consumption trends.

	National	Capital Region of Denmark	Region Zealand	The North Denmark Region	Central Denmark Region	The Region of Southern Denmark
Combinations of penicillins, including beta‐lactamase inhibitors	431 (139.4%)	341.5 (145.9%)	529.6 (52.6%)	599.9 (161.7%)	464.6 (128%)	393.3 (229%)
Penicillins	147 (48.5%)	157.6 (79.8%)	209.2 (69.8%)	192.4 (72.2%)	98.4 (−13.8%)	132.4 (62.3%)
3rd and 4th generation cephalosporins	33 (10.0%)	44.9 (69.9%)	24.9 (120.5%)	40.7 (−41.2%)	32 (−20.6%)	20.6 (30.8%)
Other	162 (8.7%)	184.9 (0.7%)	153.7 (71.2%)	204.5 (17.4%)	137.7 (51%)	140.6 (−21.2%)
Glycopeptide antibacterials	58 (−17.1%)	101.5 (−22.3%)	66 (42%)	35 (−44.2%)	40.6 (−9.5%)	26.5 (−22.9%)
Imidazole derivatives	119 (−46.6%)	155.8 (−39.7%)	191.7 (−42.9%)	86.9 (−29.5%)	27.7 (−83.2%)	148.4 (−35.6%)
Carbapenems	220 (−33.5%)	360 (−40.3%)	171.3 (0%)	198 (58.1%)	113.8 (−6.4%)	180.5 (−48.1%)
1st and 2nd generation cephalosporins	113 (−61.2%)	125 (−66.3%)	85.7 (6.2%)	181.9 (−44.8%)	90 (−58%)	100.2 (−70.2%)
Fluoroquinolones	65 (−80.4%)	78.8 (−83.2%)	111.8 (−70.5%)	86.1 (−78.2%)	56.4 (−74.7%)	24.2 (−89.4%)
Total	1348 (−20.9%)	1550.0 (−31.7%)	1543.9 (−2.6%)	1625.4 (0.3%)	1061.2 (−12.8%)	1166.7 (−25.9%)

*Note:* The table presents the antibiotic consumption in Danish ICUs in 2023, expressed as defined daily doses (DDD) per 1000 patient days, and the total relative difference in consumption from 2013 to 2023 (in parentheses) for Denmark overall and by region, categorized by antibiotic group. Positive relative values indicate increases; negative values indicate reductions. The “Total” row summarizes the overall trends across all therapeutic groups.

Abbreviations: %, percentage; DDD, defined daily dose.

### 3.2 Time Trends in Specific Antibiotic Groups

**FIGURE 1:**
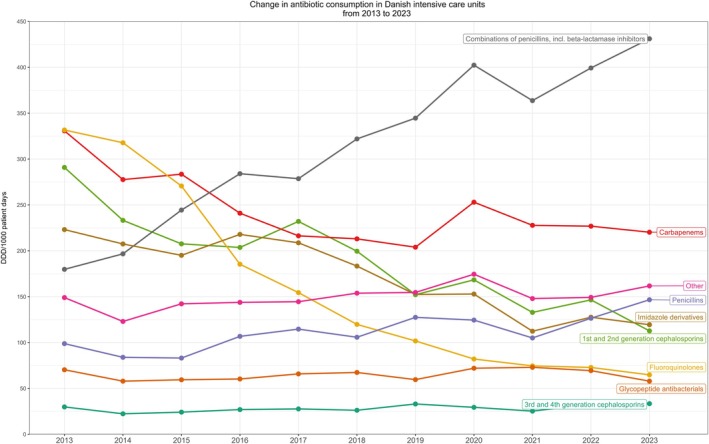
Antibiotic consumption in Danish ICUs from 2013 to 2023.

From 2013 to 2023, the consumption of fluoroquinolones declined relatively by 80%, from 332 to 65 DDD/1000 patient days (Figure 1 and S9). The use of 1st and 2nd generation cephalosporins declined by 61%, from 291 to 113 DDD/1000 patient days. Carbapenems were reduced by 34%, from 331 to 220 DDD/1000 patient days. The consumption of BL/BLI increased from 180 to 431 DDD/1000 patient days, corresponding to a 139% increase. A transient increase in consumption was observed in the calendar year 2020, coinciding with the first COVID‐19 wave. Between 2019 and 2020, carbapenem consumption rose relatively by 24% (from 204 to 253 DDD/1000 patient days), whereas BL/BLI combinations increased by 17% (from 345 to 402) before both classes resumed their downward trend in 2021–2023.

**FIGURE 2:**
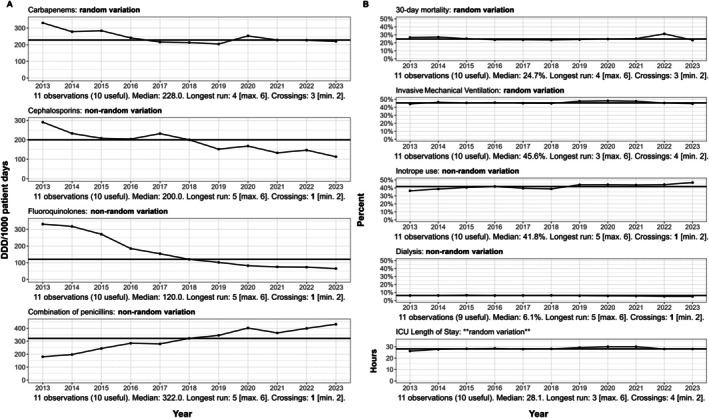
Run charts for national antibiotic consumption and key clinical outcomes in Danish ICUs, 2013–2023. Panel (A): Annual antibiotic consumption (DDD/1000 patient days) in Danish ICUs from 2013 to 2023 for four antibiotic classes—carbapenems, cephalosporins, fluoroquinolones, and BL/BLI. Each panel plots 11 observations (10 useful), with a solid horizontal line representing the overall median. The predicted upper limit for the longest run and the predicted lower limit for the number of crossings in run chart analysis are listed in brackets (e.g., “Longest run: 5 [max. 6]”). Exceeding the predicted upper limit for the longest run or falling below the predicted lower limit for crossings indicates non‐random variation at the 5% significance level. Such results are marked in bold following the plot title. DDD, defined daily doses. Panel (B): Run charts for five key patient outcomes in Danish ICUs from 2013 to 2023: 30‐day mortality, invasive mechanical ventilation, inotrope use, dialysis, and ICU length of stay. Each chart plots 11 observations (with the number of useful data points indicated) and includes a solid horizontal line marking the overall median value. The predicted upper limit for the longest run and the predicted lower limit for the number of crossings in run chart analysis are listed in brackets (e.g., “Longest run: 5 [max. 6]”). Exceeding the predicted upper limit for the longest run or falling below the predicted lower limit for crossings indicates non‐random variation at the 5% significance level. Such results are marked in bold following the plot title. The *y*‐axes display percentages for the first four outcomes and hours for ICU length of stay; the *x*‐axis denotes the year of observation.

The analyses showed that the changes in fluoroquinolones, 1st and 2nd generation cephalosporins, and BL/BLI represented non‐random variation of annual changes over time, whereas there was not sufficient evidence that changes in carbapenem consumption were due to non‐random variation. For 30‐day mortality, invasive mechanical ventilation and ICU length of stay, there was not statistically significant evidence of random variation with annual values appearing stable over time (Figure 2).

### 3.3 Variation in Antibiotic Consumption Trends Among Regions and ICUs

#### 3.3.1 Regional Variation in Consumption of Antibiotics

**FIGURE 3:**
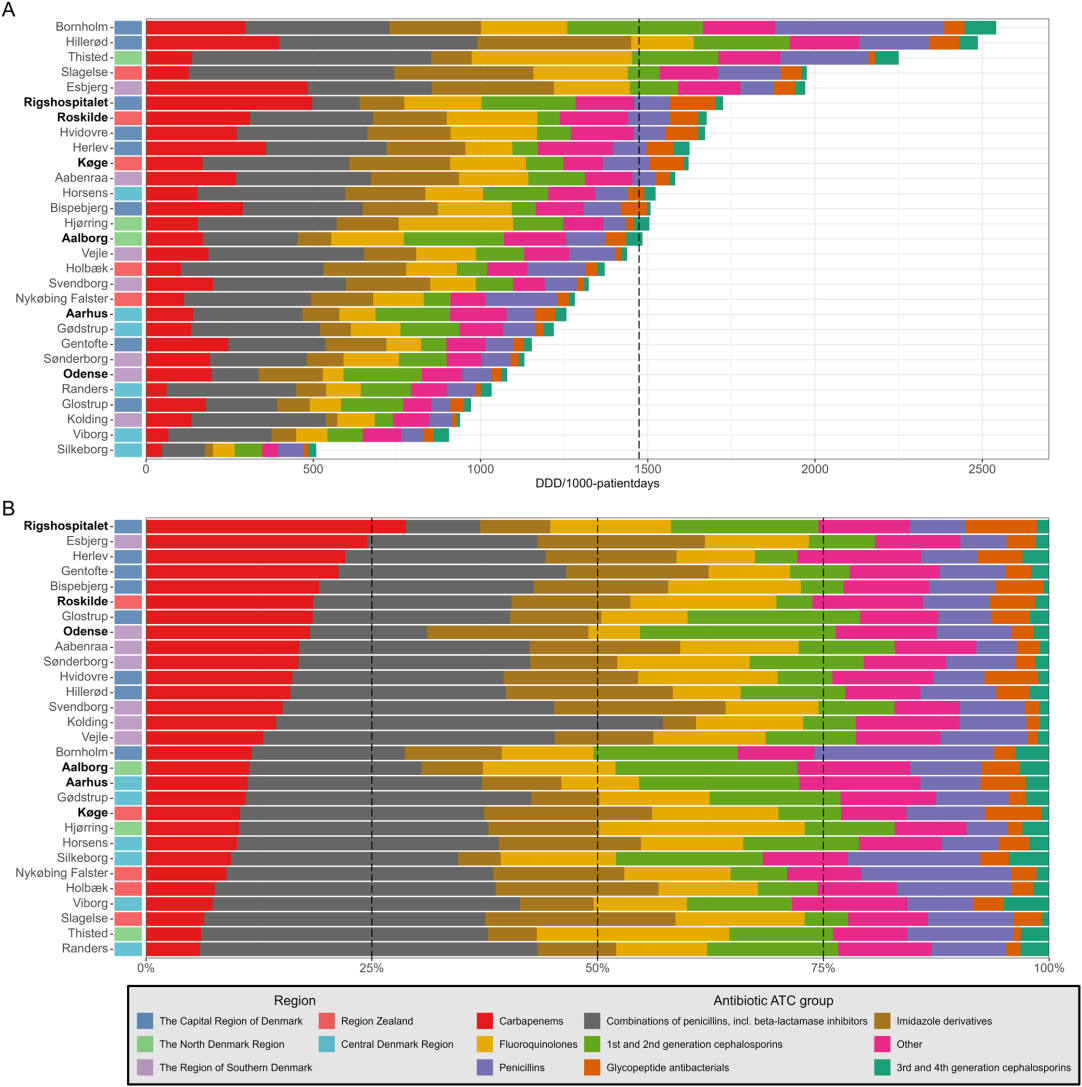
Cumulative and proportional antibiotic consumption in Danish ICUs from 2013 to 2023. Panel (A): Total antibiotic consumption in Danish ICUs from 2013 to 2023, organized by hospital and region. Bars represent cumulative consumption (DDD/1000 patient days) per hospital, segmented by antibiotic classes. Hospitals are categorized by region, indicated by color‐coded bars on the left. The dashed vertical line marks the pooled mean antibiotic consumption across all ICUs (1474 DDD/1000 patient days). University hospitals are marked in bold. See S10 and S11 for further detail. Panel (B): Proportional antibiotic consumption by Danish ICUs from 2013 to 2023, categorized by hospital and antibiotic group. Each horizontal bar represents 100% of the ICUs antibiotic use, with color‐coded segments for each antibiotic group. Hospitals are categorized by region, indicated by color‐coded bars on the left. The figure highlights variations in antibiotic use patterns across hospitals and regions over the study period. See S12 for further detail.

On the ICU‐level, antibiotic consumption ranged from 508 to 2541 DDD/1000 patient days (Figure 3a and S11).

Regional differences were seen as the consumption of carbapenems was markedly higher in the Capital Region and the Region of Southern Denmark than in the other Danish regions, but declined relatively 40% and 48%, respectively, during the study period. Taken together, carbapenems and BL/BLI accounted for approximately 44% of total antibiotic consumption each year; however, their relative contributions varied markedly by region (Figure 3b, Table 2 and S12). Usage in the Central and Zealand regions remained stable, while the North Denmark Region increased its carbapenem usage by 58% over the study period.

Fluoroquinolone consumption declined in all the regions over the study period (range of relative declines 71%–89%). Similarly, consumption of first‐ and second‐generation cephalosporins declined across regions (58%–70%).

The consumption of imidazole derivatives varied widely but declined substantially in all regions during the study period.

### 3.4 Antibiotic Consumption by the AWaRe Classification

#### 3.4.1 WHO AWaRe Classification

When applying the WHO AWaRe classification and the adapted WHO AWaRe classification, the proportional consumption of antibiotics was dominated by the ‘Watch’ category (median 76% (IQR 74%–78%) and 59% (IQR 58%–61%)), followed by the ‘Access’ (22% (IQR 21%–24%) and 22% (IQR 21%–24%)), and ‘Reserve’ categories (1% (IQR 1%–2%) and 18% (IQR 16%–20%)). The proportions of ‘Watch’, ‘Access’, and ‘Reserve’ antibiotics remained stable over the study period. The Capital Region of Denmark consumed 3%/26% of antibiotics from the ‘Reserve’ category, which is four times higher than the second highest consumer, Region Zealand, at 1%/11% (S14).

## 4 Discussion

In this nationwide study on antibiotic consumption in Danish ICUs from 2013 to 2023, we observed a substantial reduction in the overall consumption—these findings do not support our initial hypothesis of a minimal increase. This was primarily driven by a reduction in the use of fluoroquinolones, first‐ and second‐generation cephalosporins, and carbapenems. In contrast, the use of BL/BLI more than doubled in the same period. Notably, run charts showed that the changes in fluoroquinolones, cephalosporins, and penicillin combinations were statistically significantly non‐random, whereas carbapenems did not demonstrate such non‐random variation, although the results do not rule this out. The outcomes for 30‐day mortality, proportion of mechanically ventilated patients, and median ICU length of stay remained stable over the same period, suggesting no major negative impact on these outcomes. However, the complex trajectories of critically ill patients—and influences from factors outside the ICU—make it difficult to detect smaller outcome differences or attribute changes directly to altered antibiotic consumption. Moreover, crude outcome curves can mask countervailing trends. Improvements in sepsis recognition, organ‐support technology, and general ICU processes during the study period might have reduced mortality, potentially offsetting any negative impact from narrower antibiotic coverage—or vice versa. A case‐mix–adjusted metric such as the standardized mortality ratio (SMR) could have added insight; however, although SMRs are reported nationally, substantial SAPS III missingness, transition from SAPS II to SAPS 3, and source data‐flow changes make longitudinal SMR trends across 2013 to 2023 unreliable, so we report crude outcomes only.

These changes may be related to the Danish Health Authority's publication of a policy paper in November 2012, recommending prudent use of antibiotics, specifically advising that the use of carbapenems, cephalosporins, and fluoroquinolones be reduced in the Danish healthcare sector [29]. A reduction in BL/BLI consumption occurred in 2017. This may in part be explained by a nationwide back‐order from April to September 2017, and we observed a contemporaneous compensatory rise in 1st and 2nd generation cephalosporins, notably ceftazidime use. Building on this, the 2017 National Action Plan on Antibiotics in Human Healthcare set targets to reduce hospital use of critically important antibiotics by 10% by 2020 [30]. Also, the European Medicines Agency issued safety alerts on the use of fluoroquinolones in 2018 [31], and the growing availability of broad‐spectrum β‐lactams such as piperacillin–tazobactam and later‐generation cephalosporins—likely reinforced the downward shift we observed.

We were not able to determine to what extent the decline in antibiotic consumption was explained by fewer ICU patients receiving antibiotic treatment, a reduction in combination therapy, shorter treatment durations, or combinations of these.

Contrary to our observed decline, ICU antibiotic consumption in several other countries has remained stable or even increased over comparable periods. German ICUs reported a 19% overall increase in antibiotic use over a similar period, with significant relative increases in carbapenem use (+230%) and penicillin combination use (+247%) [15]. Swedish ICUs initially experienced substantial growth in antibiotic consumption but demonstrated stable use in more recent years [8, 16]. Similarly, Swiss ICUs showed a pattern of increasing consumption followed by stabilization by 2018.

The pooled mean antibiotic consumption of 1474 DDD/1000 patient days observed within Danish ICUs concurs well with data from a recent systematic review and meta‐analysis, which included 32 studies reporting ICU antibiotic consumption in 13 countries between 1997 and 2013 [5], where the pooled point estimate was found to be 1563 DDD/1000 hospital days (95% confidence interval (CI) 1472–1653). More interestingly, the point estimate for in‐ICU carbapenem consumption was found to be half of that observed in Danish and German ICUs, but the point estimate is in line with what was observed in Swiss ICUs.

In the early 2010s, an increase in Extended‐Spectrum Beta‐Lactamase (ESBL) producing Enterobacterales was observed in Denmark [32], which partly explains the guidelines recommending decreased consumption of cephalosporins. However, there could now be a need for increased use of cephalosporins as carbapenem‐sparing alternatives. Yet, a study of antibiograms across Danish ICUs could shed light on this possibility, potentially informing future guideline updates and AMS strategies.

The temporary rise in carbapenem and, to a lesser extent, BL/BLI consumption in 2020 likely reflects early‐pandemic uncertainty, when up to 70% of ICU patients with viral pneumonia received empirical broad‐spectrum antibiotics despite low rates of bacterial co‐infection (reported at 8%–14% in European cohorts) [33]. Early Danish critical care guidelines at that time recommended broad‐spectrum cover (e.g., meropenem) for suspected superinfection in mechanically‐ventilated COVID‐19 patients [34], potentially driving the spike we observed. Subsequent guideline revisions and accumulating evidence allowed prescribers to de‐escalate therapy, consistent with the rapid decline after 2020. Our dataset cannot separate COVID‐19 from non‐COVID‐19 admissions, nor account for contemporaneous changes in corticosteroid or immunomodulator use that may have influenced secondary infection risk, and we therefore interpret the pandemic‐associated fluctuation with caution.

### 4.1 Heterogeneity of Proportional Consumption of Antibiotic Classes

In the present study, total antibiotic consumption differed by as much as fivefold between ICUs, with marked variation in the relative distribution of antibiotic groups. Such variation may partly be attributed to differences in patient populations, ICU characteristics, local AMS strategies, and resistance patterns. Rates of antibiotic resistance did not seem to vary considerably between the Danish regions [35], although it is possible that antibiograms differ between ICUs. Additionally, the heterogeneity observed in antibiotic consumption suggests potential for optimization through targeted AMS programs.

These observed differences may partly stem from insufficient evidence guiding antibiotic selection. Several non‐mutually‐exclusive factors may explain the divergent carbapenem‐to‐BL/BLI ratios. First, regional clinical practice and stewardship guidance may differ interregionally. Second, microbiological epidemiology may vary between regions and even vary between ICUs intraregionally. Third, case‐mix variation—including transplant and haematology referral patterns—can shift drug choice independently of stewardship policy and influence stewardship policy. The Surviving Sepsis Campaign bases many antibiotic choice recommendations on low or very low certainty of evidence [36]. The use of metronidazole warrants further investigation, as highlighted in a recent systematic review [37]. The observed differences further highlight areas where clinical trials are needed, such as comparing broad‐spectrum antibiotics such as meropenem and piperacillin‐tazobactam [38]. Addressing these gaps could improve consistency in antibiotic prescribing and enhance AMS efforts.

### 4.2 Proportional Use of Antibiotics According to WHO AWaRe

A high proportion of antibiotics used in Danish ICUs belongs to the ‘Watch’ category in the WHO AWaRe classification. Compared to a global ICU study [39], Danish ICUs had higher ‘Watch’ use (76.1% vs. 63.0%) but lower ‘Access’ (22.4% vs. 30.6%) and ‘Reserve’ (1.4% vs. 6.4%) use. This contrasts with general Danish hospitals, where over 60% of antibiotics belong to the ‘Access’ category [35]. Despite a decline in certain antibiotic groups after the Danish Health Authority's policy on prudent use, the proportional use of ‘Watch’ and ‘Reserve’ antibiotics in ICUs remained constant, emphasizing the need for ongoing AMS efforts in ICUs.

## 5 Strengths and Limitations

The national‐level data presented here provide a robust benchmark for future investigations into antibiotic use in Danish ICUs. By capturing heterogeneity across both university and non‐university hospitals, this study establishes a valuable baseline for more targeted stewardship interventions.

These findings are nationally representative and may also apply to other high‐income countries with similar ICU infrastructures, such as the Nordic countries. However, cross‐country comparisons must consider baseline antibiotic consumption levels (e.g., DDDs/1000 patient‐days), differences in case mix, local pathogen epidemiology, and resistance patterns, which can modify both prescribing behavior and outcomes. This study can inform international comparisons and support the development of context‐specific interventions to enhance antimicrobial stewardship and patient outcomes.

Our study has some limitations. First, reliance on antibiotic sales data, while previously validated, may diverge from actual bedside administration and potentially introduces limitations due to the lack of patient‐level granularity. This precludes differentiation between empirical, prophylactic, and definitive treatments and does not allow for analyses of trends in combination therapy or treatment durations, and sales data may not reflect actual use due to stockpiling, shortages, or pandemic disruptions. Second, the DDD is a well‐established metric for antibiotic consumption, but does not necessarily reflect the prescribed daily dose [39]. The assumed average doses may underestimate requirements for obese patients and overestimate them for those with reduced renal clearance, potentially leading to misrepresentation of prescriptions. Changes in DDD definitions over the study period, such as the adjustment of meropenem from 2 to 3 g in 2019, introduce challenges in interpreting trends, although these changes in definition are accounted for in the aggregated sales data. Third, we did not have access to data on local AMS initiatives or on antimicrobial resistance patterns in Danish ICUs. Analyses on how resistance patterns affect antibiotic prescriptions and vice versa are of high importance. Fourth, a policy change in 2012 aimed at promoting prudent antibiotic use may have influenced consumption trends, but we do not have data from before this policy to compare. Fifth, we lacked case‐mix‐adjusted outcome metrics (e.g., SMR) and region‐level microbiological data (blood‐culture denominators and resistance profiles). Direct comparison with other studies also requires attention to whether those studies use patient‐days or bed‐days in the denominator and the reporting scale (DDD per 1000 vs. per 100) which does not alter trends or relative differences but complicates cross‐study comparability. Consequently, we could neither quantify the net clinical effect of changing antibiotic consumption—overall or across regions—nor explore causal links with infection epidemiology, and we could not isolate antibiotic consumption attributable to COVID‐19 ICU admissions. Despite these limitations, the findings of this study provide an essential benchmark for AMS initiatives and future research in critical care settings.

## 6 Conclusions

In this nationwide study on antibiotic consumption in Danish ICUs from 2013 to 2023, we observed a substantial reduction in overall antibiotic consumption—driven by statistically non‐random decreases in fluoroquinolones, first‐ and second‐generation cephalosporins, and other broad‐spectrum agents. During the same period, crude 30‐day mortality and ICU length of stay remained stable, although more granular, risk‐adjusted outcome analyses are needed to confirm clinical neutrality.

## Author Contributions

N.F.M., F.B.H., A.G., A.P., F.S., K.B.S., M.H.M., and M.H. conceptualized the study. N.F.M. performed the statistical analyses with help from A.G. N.F.M. and M.H. drafted the manuscript with input from the co‐authors, and all authors commented on previous versions of the manuscript. All authors read and approved the final manuscript for publication.

## Conflicts of Interest

The Department of Intensive Care at Rigshospitalet (N.F.M., A.G., A.P., and M.H.M.) has received funding for other projects from the Novo Nordisk Foundation, Sygeforsikringen ‘danmark’, the Research Council of Rigshospitalet and the Ehrenreich's Foundation and the Independent Research Fund Denmark. F.B.H. disclosed consulting roles with Gilead Sciences Denmark, Reponex Pharmaceutical, and SNIPR BIOME APS, unrelated to the current study. He also reported funding from Beta. Health and Den Frie Forskningsfond (DFF) for other projects. M.H. reported receiving research funding from AstraZeneca and travel support from Gilead and Advance Pharma. She also received honoraria from GSK, Bavarian Nordic, and Gilead for lectures and presentations, and participated on advisory boards for AstraZeneca, GSK, MSD, and Bavarian Nordic. However, none of these relationships were directly related to the content of this manuscript. K.B.S., F.S., and M.H.M. declare no conflicts of interest.

## Supporting information


**Data S1:** aas70124‐sup‐0001‐Supinfo1.

## Data Availability

The data that support the findings of this study are available from the corresponding author upon reasonable request.
