# Supplementary material for: Antibiotic Consumption in Danish Intensive Care Units, 2013–2023: A Nationwide Study of Temporal Trends
Source: Acta Anaesthesiol Scand. 2025 Sep 22;69(9):e70124. doi: 10.1111/aas.70124 (PMC12453012; doi:10.1111/aas.70124)
Supplement: Supplementary file 1 — Data S1: aas70124‐sup‐0001‐Supinfo1. [file AAS-69-0-s001.docx]

**Supplementary**

This supplementary material has been provided by the authors to give readers additional information about their work.

Supplement to:

ANTIBIOTIC USE IN DANISH INTENSIVE CARE UNITS, 2013–2023: A NATIONWIDE STUDY OF TEMPORAL TRENDS

Corresponding author:

Nick Frørup Meier, MD

Email: [nick.meier@regionh.dk](mailto:nick.meier@regionh.dk)

**Table of contents**

[ESM 1. Reporting checklist 3](#_Toc194952681)

[ESM 2. Hospital groupings 5](#_Toc194952682)

[ESM 3. Data sources 6](#_Toc194952683)

[ESM 4. WHO AWaRe and modified WHO AWaRe classification 7](#_Toc194952684)

[ESM 5. Included ATC substances 10](#_Toc194952685)

[ESM 6. ICU and hospital characteristics 13](#_Toc194952686)

[ESM 7. Admission and duration to Danish intensive care units from 2013 to 2023 16](#_Toc194952687)

[ESM 8. Proportions of missing data 20](#_Toc194952688)

[ESM 9. Figure 1: Antibiotic consumption in Danish ICUs from 2013 – 2023 23](#_Toc194952689)

[ESM 10. Relative changes in the 'Other’ group antibiotics 24](#_Toc194952690)

[ESM 11. Figure 2a: Total antibiotic consumption in Danish ICUs from 2013 to 2023 25](#_Toc194952691)

[ESM 12. Figure 2b: Proportional antibiotic consumption values 26](#_Toc194952692)

[ESM 13. Antibiotic consumption trends on regional level 28](#_Toc194952693)

[ESM 14. WHO AWaRe and adapted AWaRe analyses 29](#_Toc194952694)

[References 34](#_Toc194952695)

## ESM 1. Reporting checklist

STROBE Statement—checklist of items that should be included in reports of observational studies

STROBE Statement—Checklist of items that should be included in reports of ***cohort studies***

|  | Item No | Recommendation | Page No |
| --- | --- | --- | --- |
| Title and abstract | 1 | (*a*) Indicate the study’s design with a commonly used term in the title or the abstract | 1 |
|  |  | (*b*) Provide in the abstract an informative and balanced summary of what was done and what was found | 5 |
| Introduction | | |  |
| Background/rationale | 2 | Explain the scientific background and rationale for the investigation being reported | 7 |
| Objectives | 3 | State specific objectives, including any prespecified hypotheses | 8 |
| Methods | | |  |
| Study design | 4 | Present key elements of study design early in the paper | 8 |
| Setting | 5 | Describe the setting, locations, and relevant dates, including periods of recruitment, exposure, follow-up, and data collection | 8 |
| Participants | 6 | (*a*) Give the eligibility criteria, and the sources and methods of selection of participants. Describe methods of follow-up | 8 – 9 |
|  |  | (*b*) For matched studies, give matching criteria and number of exposed and unexposed | NA |
| Variables | 7 | Clearly define all outcomes, exposures, predictors, potential confounders, and effect modifiers. Give diagnostic criteria, if applicable | 8 – 10 |
| Data sources/ measurement | 8* | For each variable of interest, give sources of data and details of methods of assessment (measurement). Describe comparability of assessment methods if there is more than one group | 8 – 10, ESM 3 |
| Bias | 9 | Describe any efforts to address potential sources of bias | NA |
| Study size | 10 | Explain how the study size was arrived at | 8 – 9 |
| Quantitative variables | 11 | Explain how quantitative variables were handled in the analyses. If applicable, describe which groupings were chosen and why | 10 |
| Statistical methods | 12 | (*a*) Describe all statistical methods, including those used to control for confounding | 10 |
|  |  | (*b*) Describe any methods used to examine subgroups and interactions | 10 |
|  |  | (*c*) Explain how missing data were addressed | 10, ESM 8 |
|  |  | (*d*) If applicable, explain how loss to follow-up was addressed | NA |
|  |  | (*e*) Describe any sensitivity analyses | NA |

| Results | | |  |
| --- | --- | --- | --- |
| Participants | 13* | (a) Report numbers of individuals at each stage of study—e.g., numbers potentially eligible, examined for eligibility, confirmed eligible, included in the study, completing follow-up, and analysed | NA |
|  |  | (b) Give reasons for non-participation at each stage | NA |
|  |  | (c) Consider use of a flow diagram | NA |
| Descriptive data | 14* | (a) Give characteristics of study participants (e.g., demographic, clinical, social) and information on exposures and potential confounders | 11, ESM 6, ESM 7 |
|  |  | (b) Indicate number of participants with missing data for each variable of interest | 23, ESM 8 |
|  |  | (c) Summarise follow-up time (eg, average and total amount) | NA |
| Outcome data | 15* | Report numbers of outcome events or summary measures over time | 11 – 12 |
| Main results | 16 | (*a*) Give unadjusted estimates and, if applicable, confounder-adjusted estimates and their precision (eg, 95% confidence interval). Make clear which confounders were adjusted for and why they were included | 11 – 12 |
|  |  | (*b*) Report category boundaries when continuous variables were categorized | 11 – 12 |
|  |  | (*c*) If relevant, consider translating estimates of relative risk into absolute risk for a meaningful time period | NA |
| Other analyses | 17 | Report other analyses done—e.g., analyses of subgroups and interactions, and sensitivity analyses | 12 |
| Discussion | | |  |
| Key results | 18 | Summarise key results with reference to study objectives | 13 |
| Limitations | 19 | Discuss limitations of the study, taking into account sources of potential bias or imprecision. Discuss both direction and magnitude of any potential bias | 16 |
| Interpretation | 20 | Give a cautious overall interpretation of results considering objectives, limitations, multiplicity of analyses, results from similar studies, and other relevant evidence | 17 |
| Generalisability | 21 | Discuss the generalisability (external validity) of the study results | 16 |
| Other information | | |  |
| Funding | 22 | Give the source of funding and the role of the funders for the present study and, if applicable, for the original study on which the present article is based | 4 |

*Give information separately for exposed and unexposed groups.

Abbreviations: NA: Not applicable.

**Note:** An Explanation and Elaboration article discusses each checklist item and gives methodological background and published examples of transparent reporting. The STROBE checklist is best used in conjunction with this article (freely available on the Web sites of PLoS Medicine at http://www.plosmedicine.org/, Annals of Internal Medicine at http://www.annals.org/, and Epidemiology at http://www.epidem.com/). Information on the STROBE Initiative is available at http://www.strobe-statement.org.

## ESM 2. Hospital groupings

|  |  | Hospital department name |  |  |
| --- | --- | --- | --- | --- |
|  | The Capital Region of Denmark | Department of Intensive Care, 4131AN1  Department of Cardiothoracic and Cardiology intensive care, 4141+2143  Department of Neurointensive care, | ⇒ | Rigshospitalet |
|  |  | North Zealand Hospital | ⇒ | Hillerød |
|  |  | Hvidovre Hospital | ⇒ | Hvidovre |
|  |  | Herlev Hospital |  | Herlev |
|  |  | Hvidovre Hospital | ⇒ | Gentofte |
|  |  | Herlev Hospital | ⇒ | Glostrup |
|  |  | Gentofte Hospital | ⇒ | Bispebjerg |
|  |  | Glostrup Hospital | ⇒ | Bornholm |
|  | Region Zealand | Holbæk Hospital | ⇒ | Holbæk |
|  |  | Zealand University Hospital, Køge | ⇒ | Køge |
|  |  | Zealand University Hospital, Roskilde | ⇒ | Roskilde |
|  |  | Nykøbing F. Hospital | ⇒ | Nykøbing Falster |
|  |  | Slagelse Hospital | ⇒ | Slagelse |
|  | The North Denmark Region | Department of intensive care (R)  Department of intensive care (103)  Department of cardiothoracic intensive care (TIA)  Neurological and trauma intensive care unit (NOTIA) | ⇒ | Aalborg |
|  |  | Aalborg University Hospital, Thisted | ⇒ | Thisted |
|  |  | Regionhospital Nordjylland, Hjørring | ⇒ | Hjørring |
|  | Central Denmark Region | Department of intensive care (North)  Department of intensive care (East) | ⇒ | Aarhus |
|  |  | Regional Hospital, Horsens | ⇒ | Horsens |
|  |  | Regional Hospital, Randers | ⇒ | Randers |
|  |  | Regional Hospital Gødstrup  Regional Hospital Herning  Regional Hospital Holstebro | ⇒ | Gødstrup |
|  |  | Regional Hospital, Silkeborg | ⇒ | Silkeborg |
|  |  | Regional Hospital, Viborg | ⇒ | Viborg |
|  | The Region of Southern Denmark | 1. Department of intensive care (ITA1, ITA2, ITA3)  2. Department of cardiothoracic intensive care  3. Department of neurointensive care (NIA) | ⇒ | Odense |
|  |  | Svendborg Hospital | ⇒ | Svendborg |
|  |  | Esbjerg and Grindsted Hospital | ⇒ | Esbjerg |
|  |  | Lillebaelt Hospital, Kolding | ⇒ | Kolding |
|  |  | Lillebaelt Hospital, Vejle | ⇒ | Vejle |
|  |  | Hospital Sønderjylland, Aabenraa | ⇒ | Aabenraa |
|  |  | Hospital Sønderjylland, Sønderborg | ⇒ | Sønderborg |

## ESM 3. Data sources

| Variable name |  | Source |
| --- | --- | --- |
| ICU-level | ⇒ | The Register of Pharmaceutical Sales [1] |
| Hospital-level |  |  |
| Fifth-level ATC level name |  |  |
| Fourth-level ATC level name |  |  |
| Administration form |  |  |
| Dose |  |  |
| Concentration |  |  |
| Package |  |  |
| DDD |  |  |
| Admissions | ⇒ | Danish Intensive Care Database [2] |
| Admission time (in hours) |  |  |
| Case-mix numbers |  |  |
| SAPS III-score |  |  |
| Oncology (yes/no) | ⇒ | Classified according to: The Specialty plan for Clinical Oncology in Denmark [3]. |
| Haematology (yes/no) | ⇒ | Classified according to: Haematological department overview in Denmark [4]. |
| Intensive care unit beds | ⇒ | Economy and Planning departments from each respective Danish hospital |
| Hospital beds |  |  |
| WHO AWaRe classification | ⇒ | Classified according to: AWaRe classification of antibiotics for evaluation and monitoring of use, 2023 [5]. |
| WHO adapted AWaRe classification | ⇒ | Classified according to: Analysis of antibiotic use patterns in Danish hospitals 2015–2021 using an adapted version of the who aware classification [6]. |

## ESM 4. WHO AWaRe and modified WHO AWaRe classification

| Fifth-digit ATC level | Fourth-digit ATC level | WHO AWaRe classification | Adapted WHO AWaRe classification |
| --- | --- | --- | --- |
| ‘Access’ classified antibiotics | | | |
| Amikacin | Other aminoglycosides | Access | Access |
| Amoxicillin | Penicillins with extended spectrum | Access | Access |
| Amoxicillin/Clavulanic Acid | Combinations of penicillins, incl. beta-lactamase inhibitors | Access | Watch |
| Ampicillin | Penicillins with extended spectrum | Access | Access |
| Benzylpenicillin | Beta-lactamase sensitive penicillins | Access | Access |
| Cefalexin | First-generation cephalosporins | Access | Access |
| Clindamycin | Lincosamides | Access | Access |
| Cloxacillin | Beta-lactamase resistant penicillins | Access | Access |
| Dicloxacillin | Beta-lactamase resistant penicillins | Access | Access |
| Doxycycline | Tetracyclines | Access | Access |
| Flucloxacillin | Beta-lactamase resistant penicillins | Access | Access |
| Gentamicin | Other aminoglycosides | Access | Access |
| Mecillinam | Penicillins with extended spectrum | Access | Access |
| Metronidazole | Imidazole derivatives | Access | Access |
| Nitrofurantoin | Nitrofuran derivatives | Access | Access |
| Phenoxymethylpenicillin | Beta-lactamase sensitive penicillins | Access | Access |
| Pivampicillin | Penicillins with extended spectrum | Access | Access |
| Pivmecillinam | Penicillins with extended spectrum | Access | Access |
| Sulfadiazine | Intermediate-acting sulfonamides | Access | Access |
| Sulfamethizole | Short-acting sulfonamides | Access | Access |

| Sulfamethoxazole/Trimethoprim | Combinations of sulfonamides and trimethoprim, incl. Derivatives | Access | Access |
| --- | --- | --- | --- |
| Sulfametoxazol | Intermediate-acting sulfonamides | Access | Access |
| Sulfapyridine | Short-acting sulfonamides | Access | Access |
| Tetracyclin | Tetracyclines | Access | Access |
| Tigecyclin | Tetracyclines | Access | Access |
| Trimethoprim | Trimethoprim and derivatives | Access | Access |
| ‘Watch’ classified antibiotics | | | |
| Azithromycin | Macrolides | Watch | Watch |
| Cefepim | Fourth-generation cephalosporins | Watch | Watch |
| Cefotaxime | Third-generation cephalosporins | Watch | Watch |
| Ceftazidime | Third-generation cephalosporins | Watch | Watch |
| Ceftazidime | Third-generation cephalosporins | Watch | Watch |
| Ceftriaxone | Third-generation cephalosporins | Watch | Watch |
| Cefuroxime | Second-generation cephalosporins | Watch | Watch |
| Ciprofloxacin | Fluoroquinolones | Watch | Watch |
| Clarithromycin | Macrolides | Watch | Watch |
| Ertapenem | Carbapenems | Watch | Watch |
| Erythromycin | Macrolides | Watch | Watch |
| Fusidic acid | Steroid antibacterials | Watch | Watch |
| Imipenem | Carbapenems | Watch | Watch |
| Imipenem/Cilastin | Carbapenems | Watch | Watch |
| Levofloxacin | Fluoroquinolones | Watch | Watch |
| Lymecyclin | Tetracyclines | Watch | Watch |
| Meropenem | Carbapenems | Watch | Reserve |
| Methenamine | Other antibacterials | Watch | Watch |
| Moxifloxacin | Fluoroquinolones | Watch | Watch |
| Piperacillin/Tazobactam | Combinations of penicillins, incl. beta-lactamase inhibitors | Watch | Watch |
| Roxithromycin | Macrolides | Watch | Watch |
| Teicoplanin | Glycopeptide antibacterials | Watch | Watch |
| Tobramycin | Other aminoglycosides | Watch | Watch |
| Vancomycin | Glycopeptide antibacterials | Watch | Watch |
| ‘Reserve’ classified antibiotics | | | |
| Aztreonam | Monobactams | Reserve | Reserve |
| Ceftazidim/Avibactam | Third-generation cephalosporins | Reserve | Reserve |
| Ceftolozan/Tazobactam | Other cephalosporins and penems | Reserve | Reserve |
| Colistin | Polymyxins | Reserve | Reserve |
| Dalbavancin | Glycopeptide antibacterials | Reserve | Reserve |
| Daptomycin | Other antibacterials | Reserve | Reserve |
| Fosfomycin | Other antibacterials | Reserve | Reserve |
| Linezolid | Other antibacterials | Reserve | Reserve |

##

## ESM 5. Included ATC substances

|  | Third-digit ATC level | | Fourth-digit ATC level | Fifth-digit ATC level | Antibiotic group | DDD^a^ | U | Adm. route | |
| --- | --- | --- | --- | --- | --- | --- | --- | --- | --- |
|  | J01C Beta-lactam antibacterials, penicillins | | J01CA Penicillins with extended spectrum | J01CA01 ampicillin | Penicillins | 6 | g | P | |
|  | J01C Beta-lactam antibacterials, penicillins | | J01CA Penicillins with extended spectrum | J01CA11 mecillinam |  | 1.2 | g | P | |
|  | J01C Beta-lactam antibacterials, penicillins | | J01CE Beta-lactamase sensitive penicillins | J01CE01 benzylpenicillin |  | 3.6 | g | P | |
|  | J01C Beta-lactam antibacterials, penicillins | | J01CF Beta-lactamase resistant penicillins | J01CF01 dicloxacillin |  | 2 | g | P | |
|  | J01C Beta-lactam antibacterials, penicillins | | J01CF Beta-lactamase resistant penicillins | J01CF02 cloxacillin |  | 2 | g | P | |
|  | J01D Other beta-lactam antibacterials | | J01CR Combinations of penicillins, incl. beta-lactamase inhibitors | J01CR02 amoxicillin and beta-lactamase inhibitor | Combinations of penicillins, incl. beta-lactamase inhibitors | 3 | g | P |  |
|  | J01D Other beta-lactam antibacterials | | J01CR Combinations of penicillins, incl. beta-lactamase inhibitors | J01CR05 piperacillin and beta-lactamase inhibitor |  | 14 | g | P |  |
|  | J01D Other beta-lactam antibacterials | | J01DC Second-generation cephalosporins | J01DC02 cefuroxime | 1st and 2nd generation cephalosporins | 3 | g | P |  |
|  | J01D Other beta-lactam antibacterials | | J01DD Third-generation cephalosporins | J01DD01 cefotaxime | 3rd and 4th generation cephalosporins | 4 | g | P |  |
|  | J01D Other beta-lactam antibacterials | | J01DD Third-generation cephalosporins | J01DD02 ceftazidime |  | 4 | g | P |  |
|  | J01D Other beta-lactam antibacterials | | J01DD Third-generation cephalosporins | J01DD04 ceftriaxone |  | 2 | g | P |  |
|  | J01D Other beta-lactam antibacterials | | J01DD Third-generation cephalosporins | J01DD52 ceftazidime and beta-lactamase inhibitor |  | 6 | g | P |  |
|  | J01D Other beta-lactam antibacterials | | J01DE Fourth-generation cephalosporins | J01DE01 cefepime |  | 4 | g | P |  |
|  | J01D Other beta-lactam antibacterials | J01DH Carbapenems | | J01DH02 meropenem | Carbapenems | 3 | g | P | |
|  | J01D Other beta-lactam antibacterials | J01DH Carbapenems | | J01DH03 ertapenem |  | 1 | g | P | |
|  | J01D Other beta-lactam antibacterials | J01DH Carbapenems | | J01DH51 imipenem and cilastatin |  | 2 | g | P | |
|  | J01M Quinolone antibacterials | J01MA Fluoroquinolones | | J01MA02 ciprofloxacin | Fluoroquinolones | 0.8 | g | P | |
|  | J01M Quinolone antibacterials | J01MA Fluoroquinolones | | J01MA12 levofloxacin |  | 0.5 | g | P | |
|  | J01M Quinolone antibacterials | J01MA Fluoroquinolones | | J01MA14 moxifloxacin |  | 0.4 | g | P | |
|  | J01X Other antibacterials | J01XA Glycopeptide antibacterials | | J01XA01 vancomycin | Glycopeptide antibacterials | 2 | g | P | |
|  | J01X Other antibacterials | J01XA Glycopeptide antibacterials | | J01XA02 teicoplanin |  | 0.4 | g | P | |
|  | J01X Other antibacterials | J01XA Glycopeptide antibacterials | | J01XA04 dalbavancin |  | 1.5 | g | P | |
|  | J01X Other antibacterials | J01XD Imidazole derivatives | | J01XD01 metronidazole | Imidazole derivatives | 1.5 | g | P | |
|  | J01A Tetracyclines | J01AA Tetracyclines | | J01AA02 doxycycline | Other | 0.1 | g | P | |
|  | J01A Tetracyclines | J01AA Tetracyclines | | J01AA12 tigecycline |  | 0.1 | g | P | |
|  | J01A Tetracyclines | J01AA Tetracyclines | | J01AA04 lymecycline |  | 0.6 | g | P | |
|  | J01D Other beta-lactam antibacterials | J01DF Monobactams | | J01DF01 aztreonam |  | 4 | g | P | |
|  | J01D Other beta-lactam antibacterials | J01DI Other cephalosporins and penems | | J01DI54 ceftolozane and beta-lactamase inhibitor |  | 3 | g | P | |
|  | J01E Sulfonamides and trimethoprim | J01EE Combinations of sulfonamides and trimethoprim, incl. derivatives | | J01EE01 sulfamethoxazole and trimethoprim |  |  | g | P | |
|  | J01F Macrolides, lincosamides and streptogramins | J01FA Macrolides | | J01FA01 erythromycin |  | 1 | g | P | |
|  | J01F Macrolides, lincosamides and streptogramins | J01FA Macrolides | | J01FA09 clarithromycin |  | 1 | g | P | |
|  | J01F Macrolides, lincosamides and streptogramins | J01FA Macrolides | | J01FA10 azithromycin |  | 0.5 | g | P | |
|  | J01F Macrolides, lincosamides and streptogramins | J01FF Lincosamides | | J01FF01 clindamycin |  | 1.8 | g | P | |
|  | J01G Aminoglycoside antibacterials | J01GB Other aminoglycosides | | J01GB01 tobramycin |  | 0.24 | g | P | |
|  | J01G Aminoglycoside antibacterials | J01GB Other aminoglycosides | | J01GB03 gentamicin |  | 0.24 | g | P | |
|  | J01G Aminoglycoside antibacterials | J01GB Other aminoglycosides | | J01GB06 amikacin |  | 1 | g | P | |
|  | J01X Other antibacterials | J01XB Polymyxins | | J01XB01 colistin |  | 9 | MU | P | |
|  | J01X Other antibacterials | J01XC Steroid antibacterials | | J01XC01 fusidic acid |  | 1.5 | g | P | |
|  | J01X Other antibacterials | J01XX Other antibacterials | | J01XX01 fosfomycin |  | 1.2 | g | P | |
|  | J01X Other antibacterials | J01XX Other antibacterials | | J01XX08 linezolid |  | 1.2 | g | P | |
|  | J01X Other antibacterials | J01XX Other antibacterials | | J01XX09 daptomycin |  | 0.28 | g | P | |

^a^ Defined Daily Doses (DDD) as of 2024.

## ESM 6. ICU and hospital characteristics

|  | **Region** | **ICU characteristics per 2023** | | | | | | **Hospital characteristics per 2023** | |
| --- | --- | --- | --- | --- | --- | --- | --- | --- | --- |
|  |  | **Hospital** | **Intensive care unit beds,  number** | **Admissions to intensive care unit^a^, number (%)** | | | **SAPS III score^b^,  median (IQR)** | **Hospital size, number** | **Oncology/ haematology^c^,** |
|  |  |  |  | **Medical** | **Surgical admissions** | |  |  |  |
|  |  |  |  |  | **Acute** | **Elective** |  |  |  |
|  | Capital Region of Denmark | **Copenhagen University Hospital - Rigshospitalet** | 48 | 510  (13%) | 1822 (45%) | 1284  (32%) | 61  (49-73) | 905 | Yes/Yes |
|  |  | 1. Department of Intensive Care | 20 | 306  (28%) | 707  (65%) | 47  (4%) | 59  (48-73) |  |  |
|  |  | 2. Department of Cardiothoracic and Cardiology intensive care | 11 | 79  (4%) | 373  (18%) | 1237 (60%) | - |  |  |
|  |  | 3. Department of Neurointensive care | 17 | 125  (14%) | 742  (84%) | 0  (0%) | 63 (51-76) |  |  |
|  |  | North Zealand Hospital | 10 | 429  (79%) | 117  (21%) | 0  (0%) | 67 (57-76) | 572 | Yes/No |
|  |  | Hvidovre Hospital | 13 | 529  (79%) | 145  (21%) | 0  (0%) | 61 (50-70) | 633 | No/No |
|  |  | Herlev Hospital | 11 | 299  (51%) | 276  (48%) | 0  (0%) | 61 (47-72) | 709 | Yes/No |
|  |  | Gentofte Hospital | 5 | 264  (92%) | 23  (8%) | 0  (0%) | 62 (53-73) | 203 | No/No |
|  |  | Glostrup Hospital | 2 | 176  (79%) | 45  (21%) | 0  (0%) | 59 (46-70) | 114 | No/No |
|  |  | Bispebjerg Hospital | 12 | 512  (89%) | 61  (11%) | 0  (0%) | 64 (53-75) | 443 | No/No |
|  |  | Bornholm Hospital | 2 | 201  (78%) | 55  (21%) | 0  (0%) | 56 (48-70) | 74 | No/No |
|  | Region Zealand | **Zealand University Hospital, Køge** | 9 | 304  (55%) | 248  (44%) | 0  (0%) | 63 (52-74) | 400 | No/No |
|  |  | **Zealand University Hospital, Roskilde** | 6 | 364  (83%) | 57  (13%) | 14  (3%) | 65 (53-77) | 213 | Yes/Yes |
|  |  | Holbæk Hospital | 7 | 633  (87%) | 0  (0%) | 0  (0%) | 59 (49-70) | 340 | No/No |
|  |  | Nykøbing Falster Hospital | 6 | 445  (95%) | 24 (5%) | 0  (0%) | 63 (52-73) | 205 | No/No |
|  |  | Slagelse Hospital | 6 | 389  (52%) | 215  (28%) | 0  (0%) | 58 (47-71) | 338 | No/No |
|  | The North Denmark Region | **Aalborg University Hospital** | 27 | 791  (40%) | 556  (28%) | 612  (31%) | 48 (36-63) | 558 | Yes/Yes |
|  |  | 1. Department of intensive care (R) | 9 | 415  (67%) | 178  (29%) | 11  (2%) | 56 (44-67) |  |  |
|  |  | 2. Department of intensive care (103) | 5 | 286  (70%) | 79  (19%) | 41  (10%) | 48 (33-65) |  |  |
|  |  | 3. Department of cardiothoracic intensive care (TIA) | 7 | 207  (26%) | 158  (20%) | 437  (54%) | 43 (34-57) |  |  |
|  |  | 4. Neurological and trauma intensive care unit (NOTIA) | 6 | 169  (30%) | 220  (39%) | 164  (29%) | 44 (31-59) |  |  |
|  |  | Aalborg University Hospital, Thisted | 10 | 124 (100%) | 0  (0%) | 0  (0%) | 59 (51-68) | 66 | No/No |
|  |  | Regionhospital Nordjylland, Hjørring | 6 | 379  (52%) | 312  (43%) | 26  (4%) | 49 (37-62) | 19 | No/No |
|  | Central Denmark Region | **Aarhus University Hospital** | 38 | 1659 (63%) | 756  (29%) | 185  (7%) | 55 (44-66) | 850 | Yes/Yes |
|  |  | 1. Department of intensive care (North) | 18 | 921  (75%) | 263  (21%) | 38  (3%) | 52 (41-62) |  |  |
|  |  | 2. Department of intensive care (East) | 20 | 738  (53%) | 493  (35%) | 147  (11%) | 57 (47-68) |  |  |
|  |  | Regional Hospital, Horsens | 7 | 249  (51%) | 222  (45%) | 20  (4%) | - | 200 | No/No |
|  |  | Regional Hospital, Randers | 6 | 458  (62%) | 252  (34%) | 13  (2%) | 54 (44-63) | 205 | No/No |
|  |  | Regional Hospital, Gødstrup | 11 | 662  (70%) | 212  (22%) | 52  (5%) | 54 (45-66) | 557 | Yes/Yes |
|  |  | Regional Hospital, Silkeborg | 4 | 159  (92%) | 0  (0%) | 10  (6%) | 50 (42-59) | 32 | No/No |
|  |  | Regional Hospital, Viborg | 8 | 548  (62%) | 300  (34%) | 0  (0%) | 53 (43-66) | 323 | Yes/No |
|  | The Region of Southern Denmark | **Odense University Hospital** | 42 | 1287 (28%) | 1361 (31%) | 1772 (40%) | - | 623 | Yes/Yes |
|  |  | 1. Department of intensive care (ITA1, ITA2, ITA3) | 20 | 780  (39%) | 622  (31%) | 603  (30%) | - |  |  |
|  |  | 2. Department of cardiothoracic intensive care (VITA) | 12 | 419  (28%) | 381  (26%) | 664  (45%) | - |  |  |
|  |  | 3. Department of neurointensive care (NIA) | 10 | 88  (9%) | 358  (37%) | 505  (53%) | - |  |  |
|  |  | Svendborg Hospital | 6 | 208  (58%) | 129  (37%) | 14  (4%) | - | 187 | No/No |
|  |  | Esbjerg and Grindsted Hospital | 10 | 243  (60%) | 144  (35%) | 14  (3%) | 59 (48-70) | 370 | Yes/Yes |
|  |  | Lillebaelt Hospital, Kolding | 11 | 392  (56%) | 227  (33%) | 63  (9%) | 51 (38-65) | 316 | No/No |
|  |  | Lillebaelt Hospital, Vejle | 6 | 413  (85%) | 54  (11%) | 14  (3%) | 54 (43-65) | 202 | Yes/Yes |
|  |  | Hospital Sønderjylland, Aabenraa | 8 | 328  (55%) | 236  (40%) | 30  (5%) | 59 (49-71) | 302 | No/No |
|  |  | Hospital Sønderjylland, Sønderborg | 3 | 128  (79%) | 18  (11%) | 17  (10%) | 57 (45-71) | 70 | No/No |

**Legend**: The table presents the characteristics of ICUs in Denmark for 2023, categorised by region and hospital. The table includes the number of ICU beds, the distribution of admissions by medical, acute surgical, and elective surgical cases (as percentages of total admissions), and the median SAPS III scores (with IQR). Highlighted text signifies university hospital status.

^a^ Missing data for admission type ranged from 0 to 20% (ESM 7).

^b^ Missing data for SAPS III ranged from 0 to 100% (ESM 7).

^c^ Indicates whether the hospital has an oncology (yes/no) or a haematology department (yes/no) (ESM 3)

Abbreviations: IQR: interquartile range, n: number, %: percentage, SAPS III: Simplified Acute Physiology Score 3, ICU: intensive care unit

## ESM 7. Admission counts and durations to Danish intensive care units from 2013 to 2023

### Intensive care unit admissions

|  |  | **2013** | **2014** | **2015** | **2016** | **2017** | **2018** | **2019** | **2020** | **2021** | **2022** | **2023** |
| --- | --- | --- | --- | --- | --- | --- | --- | --- | --- | --- | --- | --- |
|  | **Denmark** | **30,012** | **31,808** | **30,855** | **29,008** | **29,296** | **29,094** | **27,413** | **25,829** | **27,487** | **26,402** | **26,447** |
|  | **The Capital Region of Denmark** | **7,847** | **8,423** | **7,845** | **7,090** | **7,748** | **8,130** | **8,130** | **6,582** | **7,532** | **7,429** | **7,277** |
|  | Rigshospitalet | 4,356 | 4,475 | 4,354 | 3,739 | 4,352 | 4,862 | 4,418 | 3,525 | 4,190 | 4,169 | 4,022 |
|  | Bispebjerg | 781 | 893 | 758 | 745 | 760 | 750 | 723 | 661 | 680 | 609 | 578 |
|  | Bornholm | 196 | 277 | 229 | 178 | 168 | 130 | 165 | 159 | 202 | 216 | 258 |
|  | Gentofte | 302 | 303 | 297 | 304 | 300 | 304 | 270 | 183 | 224 | 288 | 287 |
|  | Glostrup | 274 | 374 | 322 | 272 | 182 | 220 | 293 | 249 | 220 | 308 | 222 |
|  | Herlev | 614 | 657 | 595 | 606 | 620 | 594 | 638 | 589 | 596 | 573 | 581 |
|  | Hillerød | 642 | 720 | 673 | 619 | 614 | 586 | 560 | 622 | 663 | 569 | 548 |
|  | Hvidovre | 681 | 724 | 617 | 627 | 752 | 684 | 654 | 792 | 618 | 604 | 677 |
|  | **Region Zealand** | **2,958** | **3,063** | **3,028** | **2,956** | **2,992** | **2,752** | **2,516** | **2,639** | **2,898** | **2,931** | **2,947** |
|  | Holbæk | 797 | 788 | 814 | 790 | 748 | 844 | 655 | 759 | 921 | 914 | 728 |
|  | Køge | 508 | 438 | 494 | 503 | 560 | 546 | 551 | 504 | 547 | 570 | 557 |
|  | Nykøbing Falster | 581 | 597 | 579 | 631 | 638 | 458 | 538 | 594 | 554 | 533 | 470 |
|  | Roskilde | 481 | 539 | 524 | 476 | 478 | 386 | 255 | 304 | 423 | 428 | 440 |
|  | Slagelse | 592 | 701 | 617 | 556 | 568 | 518 | 517 | 478 | 453 | 486 | 752 |
|  | **The North Denmark Region** | **3,244** | **3,469** | **3,466** | **3,175** | **2,922** | **3,190** | **3,096** | **3,106** | **3,283** | **3,271** | **3,232** |
|  | Aalborg | 2,170 | 1,979 | 2,085 | 2,263 | 2,186 | 2,336 | 2,535 | 2,526 | 2,597 | 2,639 | 2,385 |
|  | Hjørring | 522 | 511 | 504 | 541 | 510 | 544 | 378 | 328 | 369 | 490 | 722 |
|  | Thisted | 204 | 222 | 232 | 211 | 152 | 204 | 183 | 152 | 144 | 142 | 125 |
|  | **Central Denmark Region** | **8,013** | **8,454** | **8,470** | **7,992** | **7,992** | **7,156** | **6,481** | **6,107** | **6,276** | **5,900** | **5,844** |
|  | Aarhus | 3,109 | 3221 | 3341 | 3090 | 3022 | 2870 | 2,922 | 2778 | 2763 | 2758 | 2617 |
|  | Viborg | 1,211 | 1178 | 1198 | 1,155 | 1250 | 1272 | 921 | 825 | 964 | 853 | 877 |
|  | Silkeborg | 287 | 421 | 372 | 244 | 216 | 180 | 201 | 149 | 132 | 116 | 173 |
|  | Horsens | 609 | 649 | 575 | 576 | 658 | 586 | 526 | 521 | 437 | 426 | 492 |
|  | Gødstrup | 1,318 | 1,341 | 1,422 | 1,364 | 1,278 | 1,184 | 1,122 | 1,047 | 1,183 | 988 | 947 |
|  | Randers | 842 | 885 | 789 | 869 | 886 | 782 | 789 | 786 | 797 | 759 | 738 |
|  | **The Region of Southern Denmark** | **7,950** | **8,399** | **8,046** | **7,795** | **7,642** | **7,866** | **7,806** | **7,395** | **7,498** | **6,871** | **7,147** |
|  | Odense | 4,160 | 4,875 | 4,558 | 4,564 | 4,358 | 4,780 | 4,952 | 4,571 | 4,667 | 4,436 | 4,446 |
|  | Svendborg | 363 | 367 | 378 | 340 | 362 | 370 | 389 | 375 | 384 | 338 | 356 |
|  | Esbjerg | 578 | 644 | 634 | 588 | 512 | 510 | 478 | 467 | 421 | 250 | 406 |
|  | Kolding | 942 | 989 | 967 | 954 | 988 | 814 | 567 | 768 | 738 | 765 | 695 |
|  | Vejle | 492 | 587 | 547 | 455 | 464 | 408 | 460 | 444 | 522 | 300 | 486 |
|  | Aabenraa | 601 | 599 | 610 | 530 | 638 | 628 | 608 | 477 | 514 | 571 | 595 |
|  | Sønderborg | 346 | 338 | 352 | 364 | 320 | 356 | 352 | 293 | 252 | 211 | 163 |

### Intensive care unit admission durations

|  |  | **2013** | **2014** | **2015** | **2016** | **2017** | **2018** | **2019** | **2020** | **2021** | **2022** | **2023** |
| --- | --- | --- | --- | --- | --- | --- | --- | --- | --- | --- | --- | --- |
|  | **Denmark** | **88** | **90** | **89** | **87** | **87** | **85** | **91** | **85** | **87** | **80** | **77** |
|  | **The Capital Region of Denmark** | **103** | **111** | **107** | **97** | **105** | **96** | **115** | **108** | **103** | **91** | **86** |
|  | Rigshospitalet | 107 | 129 | 111 | 106 | 103 | 87 | 98 | 82 | 95 | 87 | 85 |
|  | Bispebjerg | 124 | 135 | 123 | 118 | 132 | 111 | 120 | 120 | 124 | 125 | 126 |
|  | Bornholm | 44 | 44 | 38 | 39 | 46 | 54 | 26 | 39 | 34 | 34 | 31 |
|  | Gentofte | 123 | 124 | 136 | 136 | 108 | 112 | 100 | 119 | 121 | 94 | 89 |
|  | Glostrup | 101 | 87 | 87 | 124 | 116 | 91 | 93 | 60 | 127 | 69 | 73 |
|  | Herlev | 126 | 138 | 137 | 117 | 117 | 120 | 111 | 122 | 125 | 100 | 74 |
|  | Hillerød | 138 | 149 | 110 | 133 | 155 | 137 | 143 | 129 | 129 | 115 | 118 |
|  | Hvidovre | 119 | 105 | 128 | 123 | 121 | 121 | 135 | 104 | 104 | 94 | 80 |
|  | **Region Zealand** | **88** | **86** | **86** | **92** | **88** | **91** | **95** | **75** | **85** | **77** | **74** |
|  | Holbæk | 70 | 80 | 66 | 68 | 75 | 60 | 69 | 57 | 55 | 53 | 51 |
|  | Køge | 108 | 99 | 113 | 134 | 90 | 103 | 91 | 95 | 114 | 89 | 91 |
|  | Nykøbing Falster | 85 | 74 | 86 | 75 | 77 | 112 | 129 | 61 | 85 | 82 | 82 |
|  | Roskilde | 104 | 101 | 84 | 123 | 83 | 131 | 108 | 91 | 71 | 77 | 73 |
|  | Slagelse | 93 | 84 | 91 | 85 | 120 | 85 | 89 | 89 | 124 | 102 | 78 |
|  | **The North Denmark Region** | **76** | **79** | **81** | **80** | **74** | **66** | **78** | **69** | **84** | **75** | **70** |
|  | Aalborg | 81 | 91 | 95 | 82 | 70 | 67 | 73 | 68 | 83 | 77 | 75 |
|  | Hjørring | 75 | 83 | 88 | 73 | 70 | 62 | 110 | 101 | 114 | 67 | 54 |
|  | Thisted | 106 | 113 | 113 | 96 | 114 | 96 | 111 | 103 | 145 | 139 | 109 |
|  | **Central Denmark Region** | **84** | **85** | **84** | **85** | **82** | **86** | **84** | **87** | **88** | **84** | **83** |
|  | Aarhus | 85 | 90 | 86 | 81 | 89 | 81 | 93 | 98 | 105 | 96 | 99 |
|  | Viborg | 90 | 96 | 93 | 97 | 85 | 79 | 64 | 65 | 67 | 65 | 54 |
|  | Silkeborg | 211 | 116 | 148 | 223 | 251 | 317 | 295 | 281 | 302 | 397 | 264 |
|  | Horsens | 77 | 74 | 72 | 114 | 62 | 63 | 67 | 92 | 83 | 75 | 69 |
|  | Gødstrup | 70 | 73 | 71 | 63 | 66 | 78 | 63 | 69 | 63 | 53 | 65 |
|  | Randers | 63 | 61 | 72 | 72 | 55 | 57 | 59 | 53 | 57 | 52 | 52 |
|  | **The Region of Southern Denmark** | **83** | **86** | **83** | **82** | **84** | **82** | **76** | **76** | **72** | **68** | **67** |
|  | Svendborg | 107 | 118 | 80 | 97 | 116 | 122 | 107 | 105 | 94 | 84 | 74 |
|  | Odense | 84 | 71 | 77 | 91 | 78 | 76 | 73 | 74 | 64 | 55 | 59 |
|  | Esbjerg | 104 | 107 | 105 | 89 | 110 | 109 | 112 | 115 | 115 | 132 | 102 |
|  | Kolding | 91 | 87 | 83 | 101 | 90 | 93 | 101 | 92 | 95 | 89 | 88 |
|  | Vejle | 82 | 80 | 84 | 77 | 92 | 76 | 66 | 61 | 60 | 64 | 60 |
|  | Aabenraa | 62 | 68 | 60 | 64 | 53 | 66 | 60 | 66 | 77 | 80 | 68 |
|  | Sønderborg | 120 | 106 | 107 | 127 | 139 | 121 | 108 | 104 | 116 | 92 | 94 |

Admission time is in hours.

## ESM 8. Proportions of missing data

|  | **Region** | **ICU(s)** | **Admitted to ICU in 2023, number** | **Admission classification to ICU, number (%)** | **SAPS III score,  number (%)** |
| --- | --- | --- | --- | --- | --- |
|  | Capital Region of Denmark | **Rigshospitalet** | 3,812 | 406  (10%) | 2,442  (64%) |
|  |  | 1. Department of Intensive Care | 947 | 28  (3%) | 242  (26%) |
|  |  | 2. Department of Cardiothoracic and Cardiology intensive care | 1,812 | 365  (18%) | 1,812 (100%) |
|  |  | 3. Department of Neurointensive care | 831 | 13  (2%) | 319  (38%) |
|  |  | North Zealand Hospital | 513 | 0  (0%) | 13  (2%) |
|  |  | Hvidovre Hospital | 572 | 0  (0%) | 72  (11%) |
|  |  | Herlev Hospital | 559 | 5  (1%) | 6  (1%) |
|  |  | Gentofte Hospital | 268 | 0  (0%) | 19  (7%) |
|  |  | Glostrup Hospital | 222 | 0  (0%) | 70  (32%) |
|  |  | Bispebjerg Hospital | 578 | 4  (1%) | 15  (3%) |
|  |  | Bornholm Hospital | 207 | 0  (0%) | 106  (51%) |
|  | Region Zealand | Zealand University Hospital, Køge | 723 | 95  (13%) | 2  (<1%) |
|  |  | Zealand University Hospital, Roskilde | 552 | 5  (1%) | 8  (1%) |
|  |  | Holbæk Hospital | 425 | 5  (1%) | 27  (6%) |
|  |  | Nykøbing Falster Hospital | 464 | 0  (0%) | 15  (3%) |
|  |  | Slagelse Hospital | 735 | 147  (20%) | 31  (4%) |
|  | The North Denmark Region | **Aalborg University Hospital** | 2,339 | 1  (<1%) | 288  (12%) |
|  |  | 1. Department of intensive care (R) | 615 | 12  (2%) | 79  (13%) |
|  |  | 2. Department of intensive care (103) | 256 | 0  (0%) | 37  (14%) |
|  |  | 3. Department of cardiothoracic intensive care (TIA) | 802 | 0  (0%) | 45  (6%) |
|  |  | 4. Neurological and trauma intensive care unit (NOTIA) | 542 | 4  (1%) | 124  (23%) |
|  |  | Aalborg University Hospital, Thisted | 124 | 0  (0%) | 3  (2%) |
|  |  | Regionhospital Nordjylland, Hjørring | 712 | 5  (1%) | 26  (4%) |
|  | Central Denmark Region | **Aarhus University Hospital** | 2,381 | 17  (1%) | 46  (2%) |
|  |  | 1. Department of intensive care (North) | 1,206 | 3  (<1%) | 0  (0%) |
|  |  | 2. Department of intensive care (East) | 1,175 | 14  (1%) | 46  (4%) |
|  |  | Regional Hospital, Horsens | 491 | 0  (0%) | 491  (100%) |
|  |  | Regional Hospital, Randers | 728 | 15  (2%) | 25  (3%) |
|  |  | Regional Hospital, Gødstrup | 934 | 21  (2%) | 109  (12%) |
|  |  | Regional Hospital, Silkeborg | 173 | 3  (2%) | 60  (35%) |
|  |  | Regional Hospital, Viborg | 862 | 29  (3%) | 290  (34%) |
|  | The Region of Southern Denmark | **Odense University Hospital** | 4,056 | 26  (1%) | 4,056 (100%) |
|  |  | 1. Department of intensive care (ITA1, ITA2, ITA3) | 1,619 | 4  (<1%) | 1,619 (100%) |
|  |  | 2. Department of cardiothoracic intensive care (VITA) | 1,481 | 17  (1%) | 1,481 (100%) |
|  |  | 3. Department of neurointensive care (NIA) | 956 | 5  (1%) | 956 (100%) |
|  |  | Svendborg Hospital | 356 | 5  (1%) | 356 (100%) |
|  |  | Esbjerg and Grindsted hospital | 398 | 5  (1%) | 8 (2%) |
|  |  | Lillebaelt Hospital, Kolding | 692 | 13  (2%) | 452 (65%) |
|  |  | Lillebaelt Hospital, Vejle | 486 | 5  (1%) | 68  (14%) |
|  |  | Hospital Sønderjylland, Aabenraa | 591 | 0  (0%) | 1  (<1%) |
|  |  | Hospital Sønderjylland, Sønderborg | 163 | 0  (0%) | 0  (0%) |

## ESM 9. Figure 1: Antibiotic consumption in Danish ICUs from 2013 – 2023

| **Antibiotic group** | **2013** | **2014** | **2015** | **2016** | **2017** | **2018** | **2019** | **2020** | **2021** | **2022** | **2023** | **Proportional difference from 2013 to 2023**  **(DDD/1000 patient days (%))** |
| --- | --- | --- | --- | --- | --- | --- | --- | --- | --- | --- | --- | --- |
| Fluoroquinolones | 332 | 318 | 271 | 185 | 154 | 120 | 102 | 82 | 75 | 73 | 65 | -267 (-80%) |
| 1st and 2nd generation cephalosporins | 291 | 233 | 208 | 204 | 232 | 200 | 152 | 168 | 133 | 147 | 113 | -178  (-61%) |
| Imidazole derivatives | 223 | 207 | 195 | 218 | 209 | 183 | 152 | 153 | 112 | 128 | 119 | -104  (-47%) |
| Carbapenems | 331 | 278 | 284 | 241 | 216 | 213 | 204 | 253 | 228 | 227 | 220 | -111  (-34%) |
| Glycopeptide antibacterials | 70 | 58 | 59 | 60 | 66 | 67 | 60 | 72 | 73 | 69 | 58 | -12  (-17%) |
| 3rd and 4th generation cephalosporins | 30 | 22 | 24 | 27 | 28 | 26 | 33 | 29 | 25 | 33 | 33 | 3  (10%) |
| Other | 149 | 123 | 142 | 144 | 145 | 154 | 155 | 175 | 148 | 149 | 207 | 58  (39%) |
| Penicillins | 99 | 84 | 83 | 107 | 115 | 106 | 127 | 124 | 105 | 126 | 147 | (48)  (47%) |
| Combinations of penicillins, incl.  beta-lactamase inhibitors | 180 | 197 | 244 | 284 | 279 | 322 | 345 | 402 | 364 | 399 | 431 | 251  (139.4%) |
| Total | 1705 | 1520 | 1510 | 1470 | 1444 | 1391 | 1330 | 1458 | 1263 | 1351 | 1348 | -357  (-20.9%) |

Values in column 2013 through 2023 are expressed in DDD/1000 patient days.

## ESM 10. Relative changes in the 'Other’ group antibiotics

| **Other** | **Consumption of Other antibiotics in 2013 (DDD/1000 patient days (%))** | **Consumption of Other antibiotics in 2023 (DDD/1000 patient days (%))** | **Total change from 2013 to 2023 (DDD/1000 patient days (%))** |
| --- | --- | --- | --- |
| Combinations of sulfonamides and trimethoprim, incl. Derivatives | 6.1  (4.1%) | 13.6  (8.4%) | 7.5  (123%) |
| Macrolides | 48.6  (32.6%) | 97.2  (60%) | 48.6  (100%) |
| Lincosamides | 12.9  (8.7%) | 15.3  (9.4%) | 2.4  (18.6%) |
| Other antibacterials | 17.8  (11.9%) | 11.6  (7.2%) | -6.2  (-34.8%) |
| Other aminoglycosides | 47.8  (32.1%) | 17.8  (11%) | -30  (-62.8%) |
| Polymyxins | 8.2  (5.5%) | 2.2  (1.4%) | -6  (-73.2%) |
| Tetracyclines | 7.6  (5.1%) | 0.9  (0.6%) | -6.7  (-88.2%) |
| Other cephalosporins and penems | 0  (0%) | 0.4  (0.2%) | - |
| Intermediate-acting sulfonamides | 0  (0%) | 0  (0%) | - |
| Monobactams | 0  (0%) | 0  (0%) | - |
| Steroid antibacterials | 0  (0%) | 0  (0%) | - |
| Total | 149  (8.7%) | 162  (12%) | 13 (8.7%) |

## ESM 11. Figure 2a: Total antibiotic consumption in Danish ICUs from 2013 to 2023

|  | **Carbapenems** | **Combinations of penicillins, incl. beta-lactamase inhibitors** | **Imidazole derivatives** | **Fluoro-quinolones** | **1^st^ and 2^nd^ generation cephalosporins** | **Other** | **Penicillins** | **Glycopeptide antibacterials** | **3^rd^ and 4^th^ generation cephalosporins** |
| --- | --- | --- | --- | --- | --- | --- | --- | --- | --- |
| Bornholm | 298 | 430 | 273 | 258 | 406 | 215 | 506 | 61 | 94 |
| Hillerød | 397 | 593 | 460 | 187 | 288 | 208 | 209 | 91 | 54 |
| Rigshospitalet | 497 | 141 | 134 | 231 | 281 | 175 | 108 | 136 | 23 |
| Hvidovre | 271 | 390 | 249 | 258 | 101 | 187 | 95 | 100 | 20 |
| Herlev | 359 | 360 | 236 | 141 | 76 | 223 | 101 | 81 | 48 |
| Bispebjerg | 289 | 358 | 225 | 221 | 72 | 143 | 111 | 79 | 10 |
| Gentofte | 246 | 290 | 182 | 104 | 76 | 115 | 84 | 33 | 23 |
| Glostrup | 179 | 212 | 98 | 93 | 186 | 84 | 56 | 42 | 21 |
| Slagelse | 128 | 615 | 416 | 283 | 95 | 174 | 187 | 63 | 15 |
| Roskilde | 310 | 368 | 221 | 271 | 67 | 205 | 125 | 85 | 25 |
| Køge | 169 | 438 | 302 | 227 | 113 | 117 | 141 | 102 | 14 |
| Holbæk | 104 | 426 | 247 | 151 | 90 | 120 | 174 | 34 | 23 |
| Nykøbing F | 114 | 379 | 186 | 151 | 80 | 105 | 213 | 36 | 18 |
| Thisted | 138 | 714 | 121 | 479 | 258 | 185 | 264 | 19 | 71 |
| Hjørring | 155 | 415 | 185 | 342 | 150 | 120 | 69 | 24 | 45 |
| Aalborg | 170 | 284 | 100 | 218 | 299 | 185 | 118 | 63 | 48 |
| Horsens | 154 | 442 | 240 | 172 | 195 | 140 | 96 | 52 | 33 |
| Aarhus | 141 | 326 | 111 | 108 | 223 | 169 | 84 | 64 | 32 |
| Gødstrup | 135 | 385 | 92 | 149 | 177 | 129 | 98 | 24 | 31 |
| Randers | 61 | 387 | 90 | 104 | 150 | 107 | 86 | 16 | 33 |
| Viborg | 67 | 308 | 74 | 94 | 105 | 116 | 66 | 31 | 46 |
| Silkeborg | 48 | 128 | 24 | 65 | 83 | 48 | 75 | 16 | 23 |
| Esbjerg | 483 | 371 | 365 | 227 | 144 | 187 | 100 | 64 | 29 |
| Aabenraa | 269 | 403 | 264 | 207 | 169 | 143 | 70 | 40 | 16 |
| Vejle | 187 | 464 | 157 | 179 | 144 | 135 | 139 | 15 | 18 |
| Svendborg | 200 | 398 | 251 | 136 | 111 | 95 | 96 | 21 | 14 |
| Sønderborg | 191 | 290 | 109 | 166 | 143 | 103 | 87 | 25 | 17 |
| Odense | 196 | 140 | 193 | 62 | 234 | 120 | 89 | 27 | 19 |
| Kolding | 135 | 402 | 35 | 111 | 55 | 108 | 70 | 14 | 10 |

Values are expressed as DDD/1000 patient days.

## ESM 12. Figure 2b: Proportional antibiotic consumption values

|  | **Carbapenems** | **Combinations of penicillins, incl. beta-lactamase inhibitors** | **Imidazole derivatives** | **Fluoro-quinolones** | **1^st^ and 2^nd^ generation cephalosporins** | **Other** | **Penicillins** | **Glycopeptide antibacterials** | **3^rd^ and 4^th^ generation cephalosporins** |
| --- | --- | --- | --- | --- | --- | --- | --- | --- | --- |
| Rigshospitalet | 29% | 8% | 8% | 13% | 16% | 10% | 6% | 8% | 1% |
| Esbjerg | 25% | 19% | 19% | 12% | 7% | 9% | 5% | 3% | 1% |
| Herlev | 22% | 22% | 15% | 9% | 5% | 14% | 6% | 5% | 3% |
| Gentofte | 21% | 25% | 16% | 9% | 7% | 10% | 7% | 3% | 2% |
| Bispebjerg | 19% | 24% | 15% | 15% | 5% | 9% | 7% | 5% | 1% |
| Roskilde | 19% | 22% | 13% | 16% | 4% | 12% | 7% | 5% | 1% |
| Glostrup | 18% | 22% | 10% | 10% | 19% | 9% | 6% | 4% | 2% |
| Odense | 18% | 13% | 18% | 6% | 22% | 11% | 8% | 3% | 2% |
| Aabenraa | 17% | 26% | 17% | 13% | 11% | 9% | 4% | 3% | 1% |
| Sønderborg | 17% | 26% | 10% | 15% | 13% | 9% | 8% | 2% | 2% |
| Hvidovre | 16% | 23% | 15% | 15% | 6% | 11% | 6% | 6% | 1% |
| Hillerød | 16% | 24% | 18% | 8% | 12% | 8% | 8% | 4% | 2% |
| Svendborg | 15% | 30% | 19% | 10% | 8% | 7% | 7% | 2% | 1% |
| Kolding | 14% | 43% | 4% | 12% | 6% | 12% | 7% | 1% | 1% |
| Vejle | 13% | 32% | 11% | 12% | 10% | 9% | 10% | 1% | 1% |
| Bornholm | 12% | 17% | 11% | 10% | 16% | 8% | 20% | 2% | 4% |
| Aalborg | 11% | 19% | 7% | 15% | 20% | 12% | 8% | 4% | 3% |
| Aarhus | 11% | 26% | 9% | 9% | 18% | 13% | 7% | 5% | 3% |
| Gødstrup | 11% | 32% | 8% | 12% | 15% | 11% | 8% | 2% | 3% |
| Køge | 10% | 27% | 19% | 14% | 7% | 7% | 9% | 6% | 1% |
| Hjørring | 10% | 28% | 12% | 23% | 10% | 8% | 5% | 2% | 3% |
| Horsens | 10% | 29% | 16% | 11% | 13% | 9% | 6% | 3% | 2% |
| Silkeborg | 9% | 25% | 5% | 13% | 16% | 9% | 15% | 3% | 4% |
| Nykøbing F | 9% | 30% | 15% | 12% | 6% | 8% | 17% | 3% | 1% |
| Holbæk | 8% | 31% | 18% | 11% | 7% | 9% | 13% | 2% | 2% |
| Viborg | 7% | 34% | 8% | 10% | 12% | 13% | 7% | 3% | 5% |
| Slagelse | 6% | 31% | 21% | 14% | 5% | 9% | 9% | 3% | 1% |
| Thisted | 6% | 32% | 5% | 21% | 11% | 8% | 12% | 1% | 3% |
| Randers | 6% | 37% | 9% | 10% | 15% | 10% | 8% | 2% | 3% |

## ESM 13. Antibiotic consumption trends on regional level


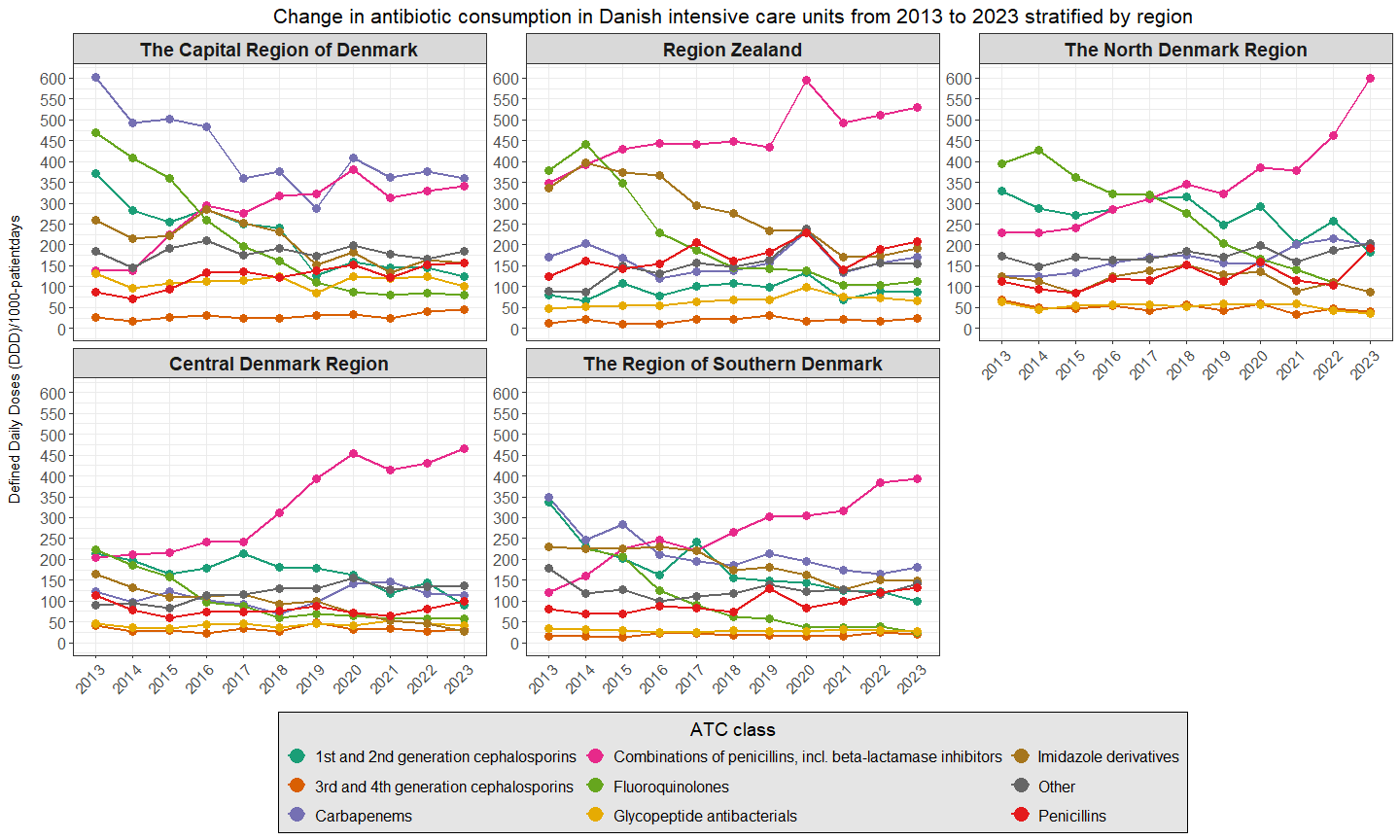


## ESM 14. WHO AWaRe and adapted AWaRe analyses

###
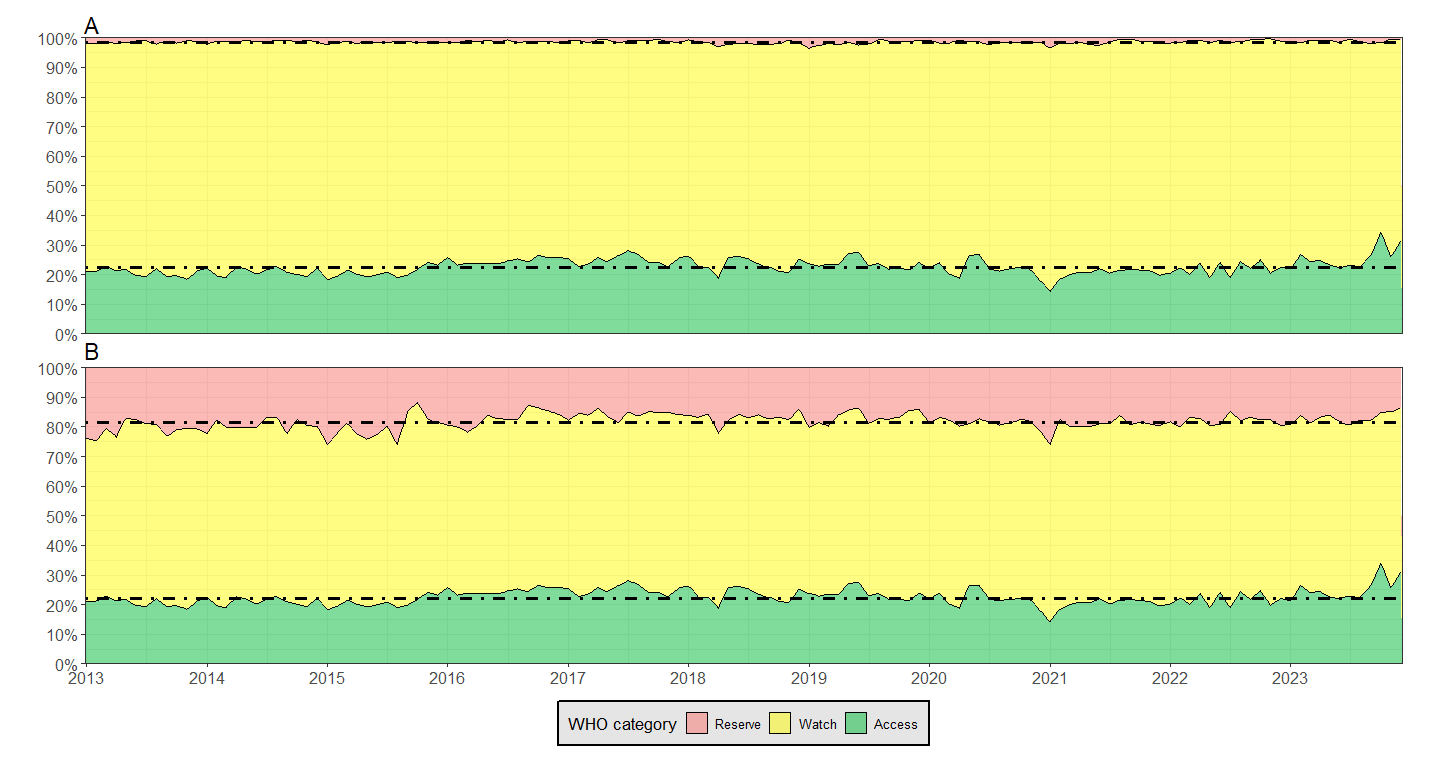
Proportional WHO AWaRe antibiotic consumption in Danish ICUs from 2013 to 2023

**Legend**: Proportional antimicrobial consumption in Danish ICUs from 2013 to 2023, categorised by the WHO AWaRe classification (Panel A) and an adapted version (Panel B). Proportions of ‘Access’ (green), ‘Watch’ (yellow), and ‘Reserve’ (red) antimicrobials are shown over time. Panel A: Standard WHO AWaRe classification; ‘Watch’ group consistently dominates consumption, followed by ‘Access’, with minimal ‘Reserve’ use. Panel B: Adapted classification with reclassified antimicrobials, resulting in increased ‘Reserve’ and decreased ‘Watch’ proportions. Dashed lines represent median consumption of ‘Access’ (lower line) and ‘Watch’ (upper line) antimicrobials. Both panels display stable patterns.

### WHO AWaRe classification distribution on regional level


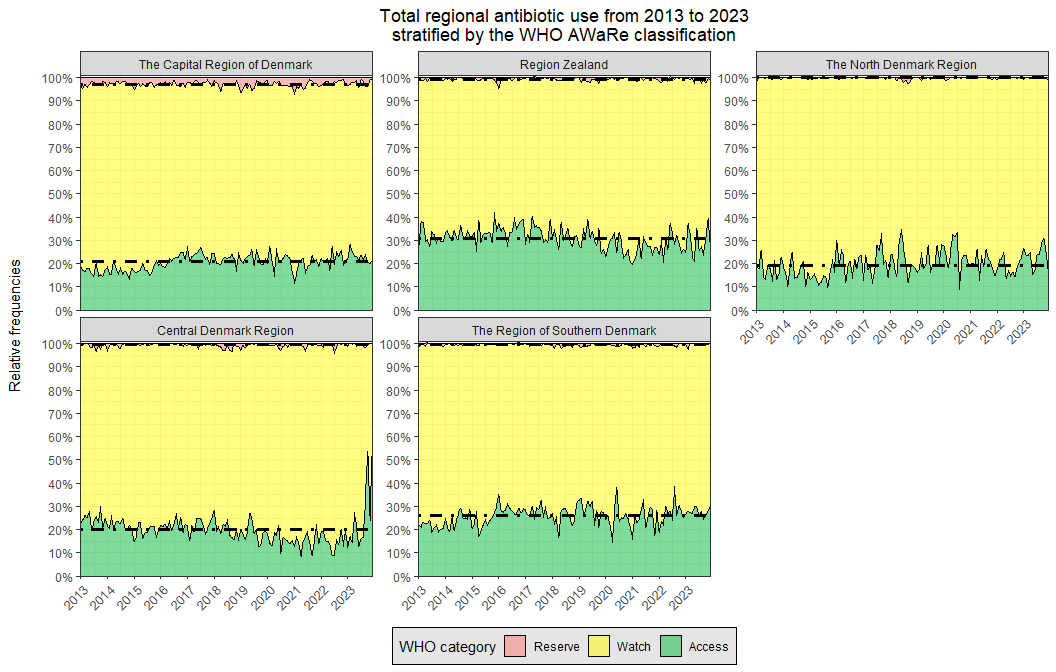


| **Region** | **Access** | **Watch** | **Reserve** |
| --- | --- | --- | --- |
| The Capital Region of Denmark | 20.9% | 76.3% | 2.8% |
| Region Zealand | 30.8% | 68.5% | 0.7% |
| The North Denmark Region | 19.3% | 80.5% | 0.2% |
| Central Denmark Region | 19.9% | 79.7% | 0.4% |
| The Region of Southern Denmark | 26.2% | 73.3% | 0.1% |

Values are expressed as median percentage of antibiotic consumption in the categories of Access, Watch, and Reserve over the 11-year period [5].

### Adapted WHO AWaRe classification distribution on regional level


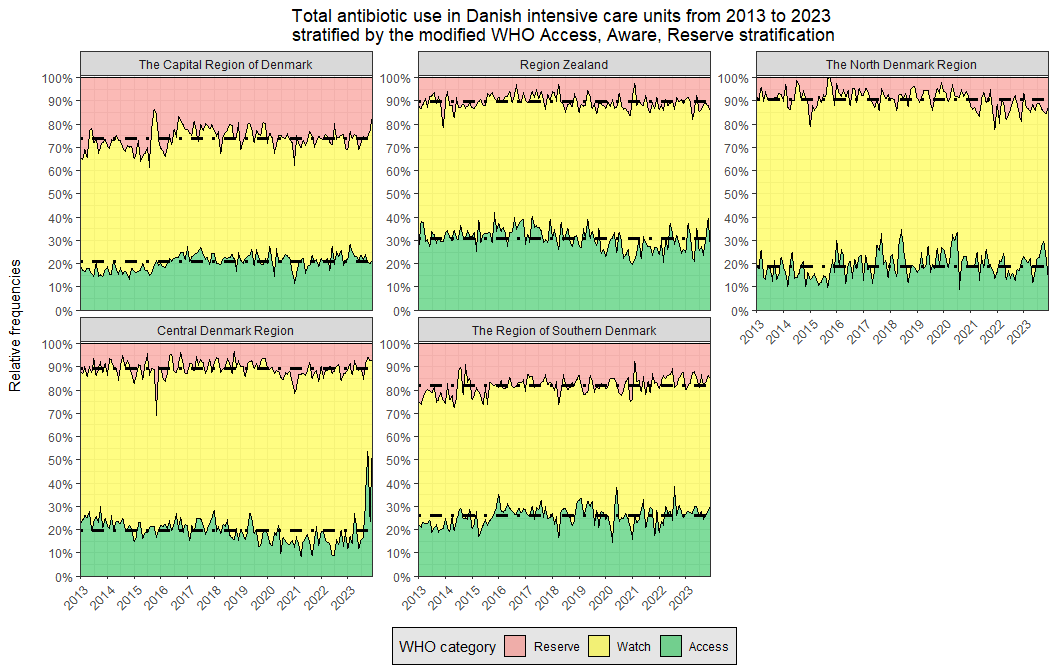


| **Region** | **Access** | **Watch** | **Reserve** |
| --- | --- | --- | --- |
| The Capital Region of Denmark | 20.9% | 52.9% | 26.1% |
| Region Zealand | 30.8% | 58.7% | 10.9% |
| The North Denmark Region | 18.9% | 71.5% | 8.8% |
| Central Denmark Region | 19.6% | 69.5% | 10.7% |
| The Region of Southern Denmark | 26.2% | 55.8% | 18.0% |

Values are expressed as median percentage of antibiotic consumption in the categories of Access, Watch, and Reserve over the 11-year period [6].

## References

1. Wallach Kildemoes H, Toft Sørensen H, Hallas J. The Danish National Prescription Registry. Scand J Public Health. 2011 Jul;39(7_suppl):38–41.

2. Christiansen CF, Møller MH, Nielsen H, Christensen S. The Danish Intensive Care Database. Clin Epidemiol. 2016 Oct;Volume 8:525–30.

3. Specialerapport-for-Klinisk-onkologi.pdf [Internet]. [cited 2025 Jan 9]. Available from: https://sst.dk/-/media/Viden/Specialplaner/Specialeplan-for-klinisk-onkologi/Specialerapport-for-Klinisk-onkologi.ashx

4. Afdelinger [Internet]. Dansk Hæmatologisk Selskab. [cited 2025 Jan 9]. Available from: https://hematology.dk/afdelinger

5. AWaRe classification of antibiotics for evaluation and monitoring of use, 2023 [Internet]. [cited 2025 Jan 9]. Available from: https://www.who.int/publications/i/item/WHO-MHP-HPS-EML-2023.04

6. Anhøj J, Boel J, Olesen BR, Bak HB, Hellesøe AMB, Thomsen K, et al. Analysis of antibiotic use patterns in Danish hospitals 2015–2021 using an adapted version of the who aware classification. BMJ Open Qual. 2022 Nov;11(4):e002098.
